# Alterations in sea urchin (*Mesocentrotus nudus*) microbiota and their potential contributions to host according to barren severity

**DOI:** 10.1038/s41522-023-00450-z

**Published:** 2023-10-31

**Authors:** Joon-Young Park, Jae-Won Jo, Yu-Jeong An, Jin-Jae Lee, Bong-Soo Kim

**Affiliations:** 1 Department of Life Science, Multidisciplinary Genome Institute, Hallym University, Chuncheon, Gangwon-do 24252 Republic of Korea; 2 The Korean Institute of Nutrition, Hallym University, Chuncheon, Gangwon-do Republic of Korea

**Keywords:** Microbiome, Metagenomics

## Abstract

Sea urchins are biotic factors driving the decline of kelp forests in marine ecosystems. However, few studies have analyzed the microbiota of surviving sea urchins in barren regions with scarce diet resources. Here, we analyzed the microbiota in the pharynx and gut of the sea urchin *Mesocentrotus nudus* located along the coast of an expanding barren region in South Korea. The ecological adaptation of genera in sea urchins was predicted using the neutral assembly model. The pharynx and gut microbiota were different, and microbes in the surrounding habitats dispersed more to the pharynx than to the gut. The gut microbiota in sea urchins is altered by barren severity and plays different roles in host energy metabolism. These findings help to understand the microbiota in sea urchins according to urchin barren and its contribution to the survival of sea urchins in severe barren regions with limited macroalgae.

## Introduction

Kelp forests (macroalgal forests), which account for 25% of the world’s coastline, are important in marine ecosystems because they provide habitats and nutrients for a diverse array of organisms^1,2^. Kelp forests are biodiversity hotspots in marine ecosystems and can be used as sentinels of ecological change, considering their high responsiveness to environmental conditions^3,4^. In recent decades, a frequent decline in kelp forests has been observed worldwide. Various factors, including coastal warming, eutrophication, kelp herbivores, and disease, can cause kelp deforestation^5–8^. At high latitudes, the sea urchin has been the most common agent, leading to ecosystem shifts from kelp-dominated to urchin-dominated state, called urchin barrens^9,10^. The overharvest of sea urchin predators has been linked to the high density of sea urchins^11^. Sea urchins can eliminate macroalgae and maintain the barrens^10,12^. Therefore, abrupt shifts from kelp forests to urchin barren stable states make it difficult to reverse phase changes and entail an ecosystem function loss^13^. Restoration efforts have been conducted in several countries, but an effective solution to relieve the damage caused by the urchin barren has not been reported yet^14–16^. The area of the urchin barren on the eastern coast of South Korea has expanded from 2413 ha in 2003–2004 to 10,518 ha in 2014^17^. Sea urchins are a major factor in the expansion of barren owing to a significant correlation between macroalgal abundance and sea urchin density on the eastern coast of South Korea^18^. The South Korean government has been making efforts to restore marine ecosystems in these barren regions since 2009^19^.

The sea urchin is an important aquatic resource that plays a positive role in the coral reef ecosystem by controlling the overgrowth of algae by feeding on them^20,21^. However, high densities of sea urchins can overgraze reef and kelp forests, causing barren states in subtidal habitats^22,23^. Sea urchin populations can prevent macroalgal recovery even after the depletion of algae resources, and algae begin to grow after the removal of sea urchins by urchin predators^10,24^. Various studies have been conducted to understand the behavioral characteristics of sea urchins and their relationship with environmental changes during the restoration of kelp forests^25–27^. Although removing sea urchins can be a simple and effective method for promoting kelp recovery, it does not provide a long-term solution to restore kelp ecosystems^28^. Therefore, a comprehensive understanding of sea urchins within urchin-barren regions is necessary to strategically restore kelp ecosystems.

Microbes dynamically interact with their hosts and play important roles in the metabolism, immunity, and development of hosts^29,30^. Recently, several studies have reported the structure, function, and dynamics of gut microbiome in sea urchins^31–35^. The composition of gut microbiota varies according to the sea urchin species, and nitrogen-fixing bacteria in the gut promote the growth of sea urchins by supplying nitrogen to the host. Although the feeding behavior of sea urchins has been studied to understand ecological interactions in kelp deforest area^22,36^, few studies have analyzed the microbiota of sea urchins in these regions to understand the potential roles of microbiota for surviving in depleted preferred diet resources. Recently, combination methods of sea urchin removal with other managements, including marine protection and predator protection, are suggested as more effective restoration methods^28^. Understanding the role of microbiome in sea urchins can provide ecological insights to apply.

In this study, we analyzed the microbiota in the pharynx and gut of *Mesocentrotus nudus*, a common sea urchin along the coast of South Korea, and the microbiota of their habitats (sand and seawater) collected from eight barren regions (five mild and three severe barren regions) in South Korea. The ecological dynamic of microbiota in sea urchins was predicted by the neutral assembly model to identify ecologically adapted microbes^37^. This study provides novel insights into the microbiota of sea urchins based on the neutral assembly model and expands our understanding of microbiota in sea urchins according to diet availability by kelp deforestation. Further studies, including longitudinal studies with various meta-omics, could advance the ecological concept of phase shifts between forested and urchin barren stable states with host-microbiome interactions.

## Results

### Microbiota is different between the pharynx and gut of sea urchin as well as in their habitats

**Fig. 1:**
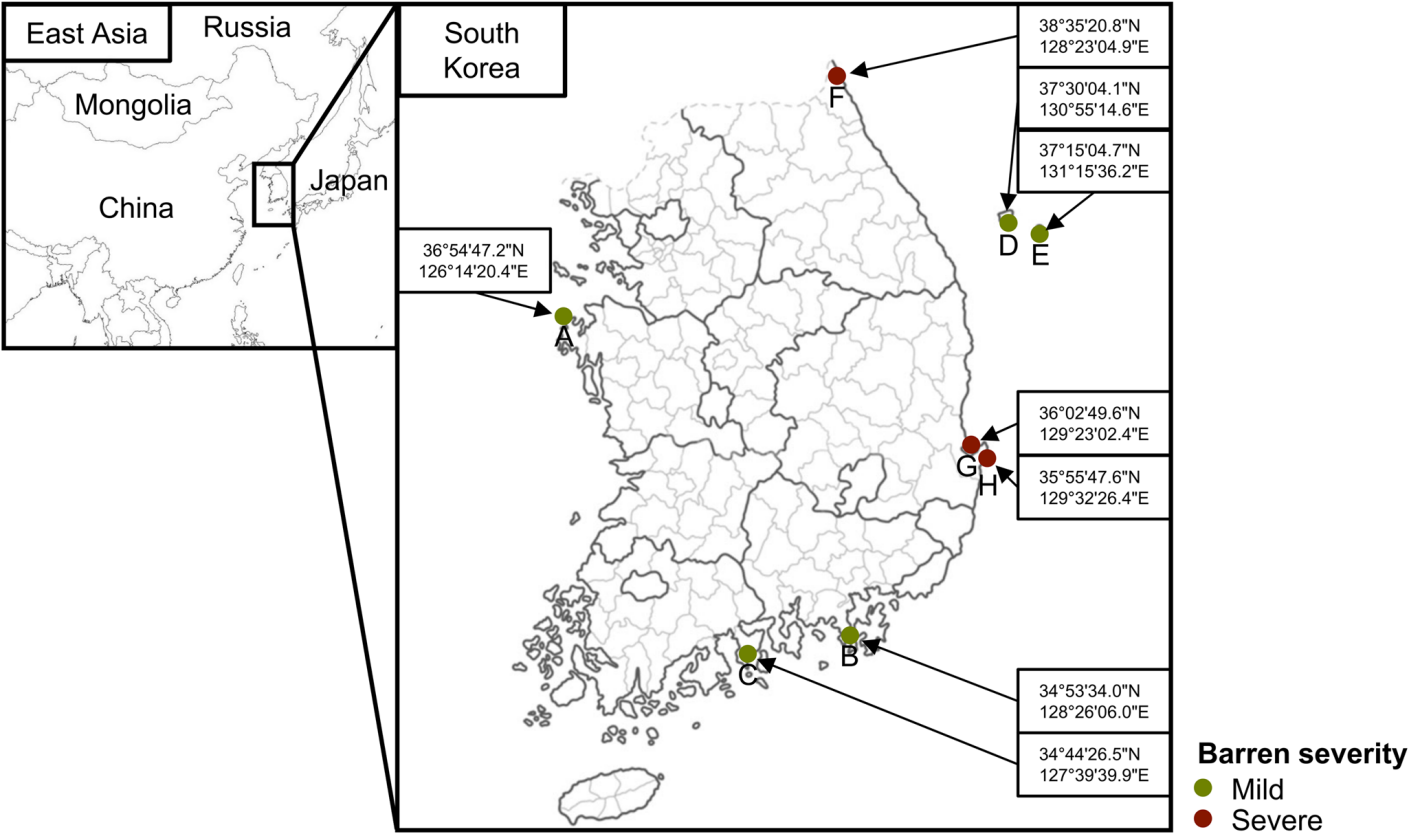
Sampling sites of sea urchins. Samples were collected from five mild barren regions (grass green circles; **A**–**E**) and three severe barren regions (brown circles; **F**, **G**, and **H**). Sampling sites were selected based on the annual report about population dynamics data of sea urchins and the survey report of urchin barren located in the South Korean coast. **A**, Taean; **B**, Tongyoung; **C**, Yeosoo; **D**, Ulleng do; **E**, Dokdo; **F,** Goseong; **G**, Homigot; **H**, Gooryongpo.

Sea urchins (*n* = 7 at each site) were randomly collected from five mild barren regions and three severe barren regions in South Korea (Fig. 1 and Supplementary Table 1). Negative controls at every step were checked and sequenced along with the samples because potential contaminants could produce biased results in sequencing-based studies using low-biomass samples^38,39^. One sea urchin sample in each site (except site G) was excluded owing to failed sequencing library preparation. Thus, a total of 105 sequence data obtained from samples of sea urchins (49 pharynx and 49 gut) and habitats (3 seawater and 4 sand) were analyzed after removing potential contaminants based on sequences detected in 27 negative controls (from sampling to library preparation processes). The microbiota significantly differed between collected samples and negative controls (PERMANOVA test, *p* < 0.001; Supplementary Figure 1). Potential contaminant sequences were trimmed based on detected sequences in negative controls according to the Decontam pipeline (Supplementary Table 2).

**Fig. 2:**
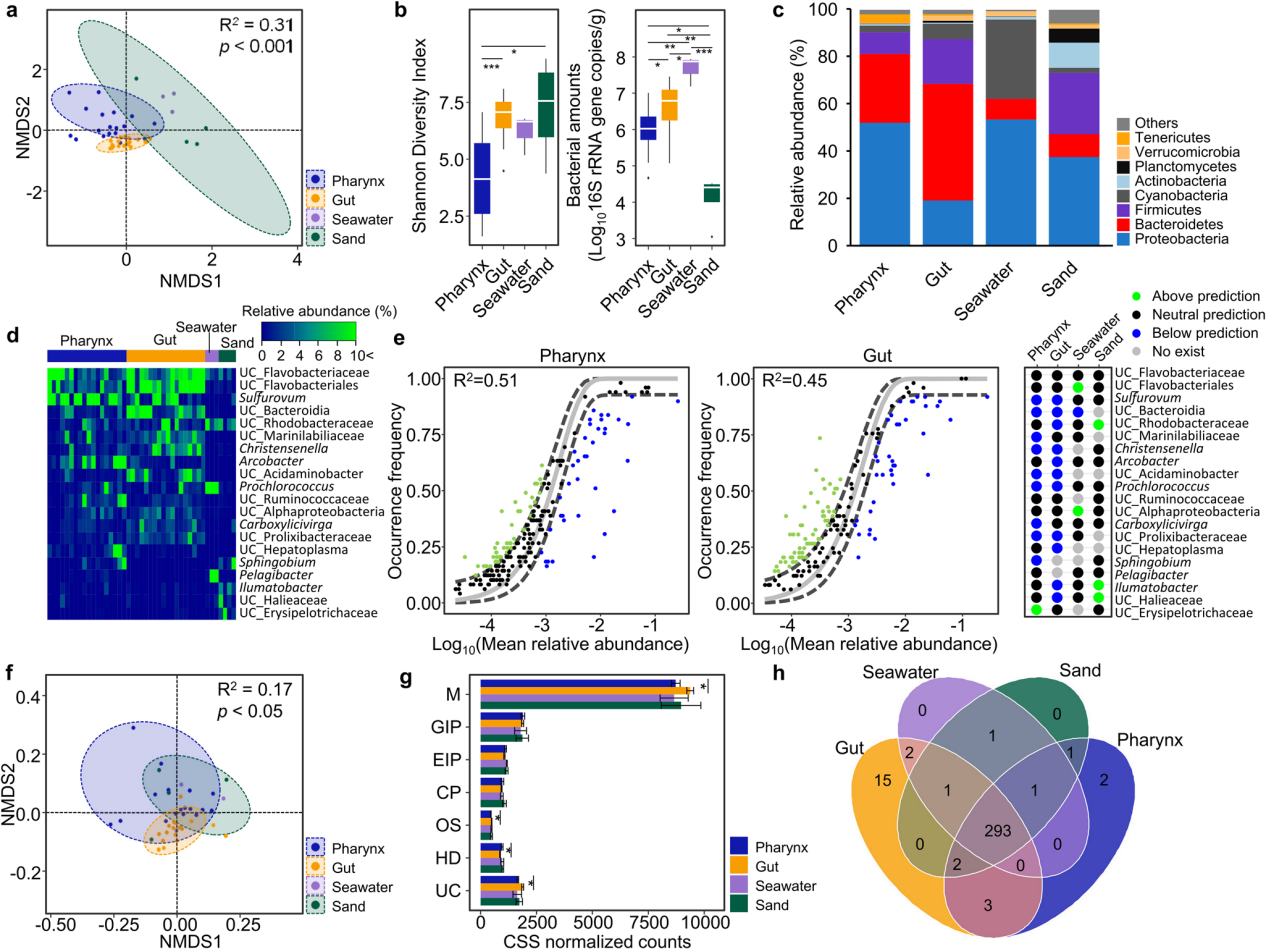
Comparison of microbiota among samples. **a** Bacterial compositions in the pharynx (*n* = 49) and gut (*n* = 49) of sea urchins, seawater (*n* = 3), and sand (*n* = 4) were compared using the NMDS plot. The *p* value was calculated using PERMANOVA. **b** The diversity of microbiota and bacterial amounts were compared among samples. Bounds of boxes represent the first quartiles (Q1) and the third quartiles (Q3), center lines represent the median values, and the whiskers stretch to 1.5 times. The significance was adjusted using Benjamini-Hochberg false discovery rate (FDR) multiple testing correction with Dunn’s test. *q < 0.05, **q < 0.01, ***q < 0.001. **c** Composition of the microbiota was compared among samples at the phylum level. Phyla with relative abundance < 1% in every sample group were combined with the “others”. Bar plots show the mean relative abundances of phyla in each group. **d** Dominant genera in each sample group were compared using the heatmap analysis. Different colors indicate the relative abundance of each genus. **e** Comparison of fit to the neutral model among samples. The neutral model plots show predicted occurrence frequencies for the pharynx and gut microbiota. Genera detected more frequently than that predicted by the model are shown in light green, while genera detected less frequently than that predicted are shown in blue. Dashed lines represent 95% CIs around the model prediction (gray line). The comparison of dominant genera deviated from the neutral model among samples. Light green circles indicate above the neutral prediction, blue circles indicate below prediction, black circles indicate in the prediction, and light gray circles indicate no genus detected in the sample. **f** Compositions of predicted functional features at the KEGG 3rd category level were compared among samples in the NMDS plots. **g** Averages of normalized counts for each KEGG 1st category level were compared among samples. Bar plots show mean ± S.D. The significance was calculated using the Wilcoxon-rank sum test. M Metabolism; GIP Genetic Information Processing; EIP Environmental Information Processing; CP Cellular Processes; OS Organismal Systems; HD Human Diseases; UC Unclassified. **h** The number of shared functional features between samples was shown in the Venn diagram.

The microbiota of sea urchins was different from that of their habitats (sand and seawater), also differing between the pharynx and gut of the sea urchins (PERMANOVA test, *p* < 0.001; Fig. 2a). Bacterial diversity was higher in the gut than in the pharynx (Dunn’s test, *q* < 0.001), but diversity in the sand was highest among the samples (Fig. 2b). Bacterial amounts were also higher in the gut than in the pharynx (Dunn’s test, *q* < 0.05). The lowest bacterial amounts were detected in sand samples. Proteobacteria was the predominant phylum in the pharynx of sea urchins, seawater, and sand samples. In contrast, Bacteroidetes was predominant in the gut of sea urchins (Fig. 2c). The dominant genus composition (selected by a prevalence of >30% and relative abundance >0.05% in each group) was different between the sea urchin and habitat samples (sand and seawater; Fig. 2d). Although *Sulfurovum*, uncultured (UC)_Flavobacteriaceae, and UC_Flavobacteriales were the dominant genera in the pharynx and gut, the relative abundance of other dominant members was different between these two organs. *Prochlorococcus*, UC_Rhodobacteraceae, UC_Flavobacteriaceae, and *Pelagibacter* were the dominant genera in seawater, whereas UC_Erysipelotrichaceae, UC_Flavobacteriaceae, *Sphingobium*, and *Illumatobacter* were the dominant genera in sand samples. The microbiota in the surrounding habitats could be transferred to sea urchins through feeding activity. Thus, the dispersal of dominant genera from habitats to the pharynx and gut of the sea urchins was predicted using a neutral assembly model (Fig. 2e). Points that differ significantly from the neutral prediction indicate that the genera are actively selected for or against according to the host or habitat condition. The model had a better fit for the microbiota in the pharynx (56.9% of detected genera fit the model; R^2^ = 0.51) than for the microbiota in the gut (42.5% of genera fit the model; R^2^ = 0.45). Ten dominant genera from the microbiota of the pharynx and 11 from that of the gut in the sea urchins deviated from the neutral prediction. *Sulfurovum*, UC_Bacteroidia, *Christensenella*, UC_Acidaminobacter, *Prochlorococcus*, and UC_Prolixibacteraceae were detected in both the pharynx and gut as ecologically adapted genera in the sea urchin body. However, 10 genera in the pharynx and 7 genera in the gut were predicted to be neutral dispersal microbes from the microbiota in their habitats. In particular, UC_Flavobacteriaceae and UC_Flavobacteriales were dominant members with high relative abundance in both the pharynx and gut, but they could be dispersal microbes between habitats.

The predicted functions of the microbiota were also significantly different among samples (PERMANOVA test, *p* < 0.05; Fig. 2f). The composition of KEGG Orthology (KO) at the 1st category level was compared among samples, and the metabolism was the predominant category in all samples (Fig. 2g). However, the predicted functions of the microbiota were different between the pharynx and gut. The metabolism, organismal systems, and unclassified categories were higher in the gut than in the pharynx, whereas the human disease category was higher in the pharynx (Wilcoxon-rank sum test, *p* < 0.05). At the 3rd category level, 293 KOs were common in all samples, 298 KOs were common in the pharynx and gut of sea urchins, and 20 KOs were unique in sea urchin samples (Fig. 2h). The number of predicted microbiota functions was higher in the gut and pharynx of sea urchins than in their habitat samples.

### Environmental variables, more than individual growth, influence sea urchin microbiota

Environmental conditions and host factors influence the microbiota in sea urchins as well as the microbiota in the surrounding environments. A previous study reported an association between the microbiota and sea urchin growth stage (body weight and shell length)^35^. Therefore, we analyzed whether environmental or host growth factors are strongly associated with the microbiota in the pharynx or gut using the EnvFit model (Supplementary Table 3). The microbiota in sea urchins was influenced more by environmental factors than by the growth of host. Regional differences and seawater temperature were significantly associated with variations in microbiota in both the pharynx and gut (PERMANOVA and Mantel test, *p* ≤ 0.003). Barren severity was only significantly associated with gut microbiota (PERMANOVA test, R^2^ = 0.223, *p* = 0.001). Although the weight and diameter of sea urchins were associated with pharynx microbiota (Mantel test, *p* < 0.05), host growth was not associated with gut microbiota. Seawater temperature and barren severity are components of regional differences. Thus, we analyzed the correlation between seawater temperature and microbiota first.

**Fig. 3:**
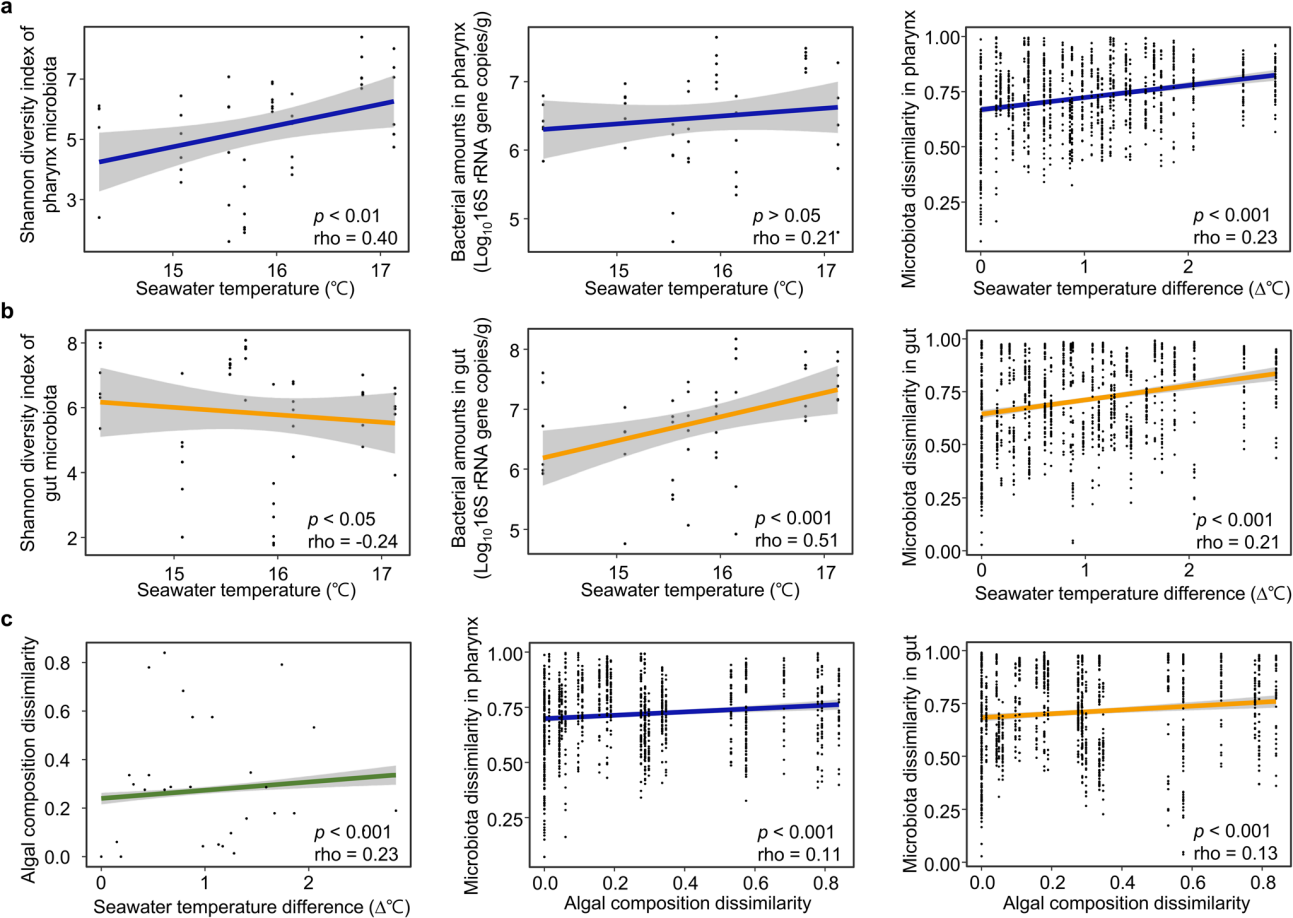
Correlations between microbiota and seawater temperature were compared between the pharynx and gut of sea urchins. **a** The microbiota diversity in the pharynx was positively correlated with seawater temperature, whereas bacterial amounts were not significantly correlated with seawater temperature. Microbiota dissimilarities in the pharynx were correlated with differences in seawater temperature. **b** The microbiota diversity in the gut was negatively correlated with seawater temperature, whereas bacterial amounts were positively correlated with seawater temperature. Microbiota dissimilarities in the gut were correlated with differences in seawater temperature. **c** Dissimilarity of algal composition was correlated with differences in seawater temperature. Dissimilarities in the microbiota of the pharynx and gut were correlated with dissimilarities of algal composition. The dissimilarity of composition was based on the Bray-Curtis distance. The correlation and significance were determined using the Spearman correlation analysis.

The diversity of the microbiota in the pharynx was positively correlated with seawater temperature (Spearman correlation, *p* < 0.01), and the microbiota dissimilarity was correlated with seawater temperature differences (*p* < 0.001; Fig. 3a). However, the microbial diversity in the gut was negatively correlated with seawater temperature (*p* < 0.05), and bacterial amounts increased as the seawater temperature increased (*p* < 0.001; Fig. 3b). Microbiota dissimilarity in the gut was also correlated with seawater temperature differences (*p* < 0.001). Seawater temperature could affect the algal composition at each sampling site, and the algal composition could influence the microbiota in sea urchins through feeding. Thus, we analyzed these associations based on the algal composition at each site (Supplementary Table 3). The difference in algal composition was correlated with seawater temperature, and these differences were related to microbial dissimilarity in both the pharynx and gut (*p* < 0.001; Fig. 3c). The correlation between algal composition and microbiota differences was higher in the gut (Spearman correlation, rho = 0.13, *p* < 0.001) than in the pharynx (rho = 0.11, *p* < 0.001).

**Fig. 4:**
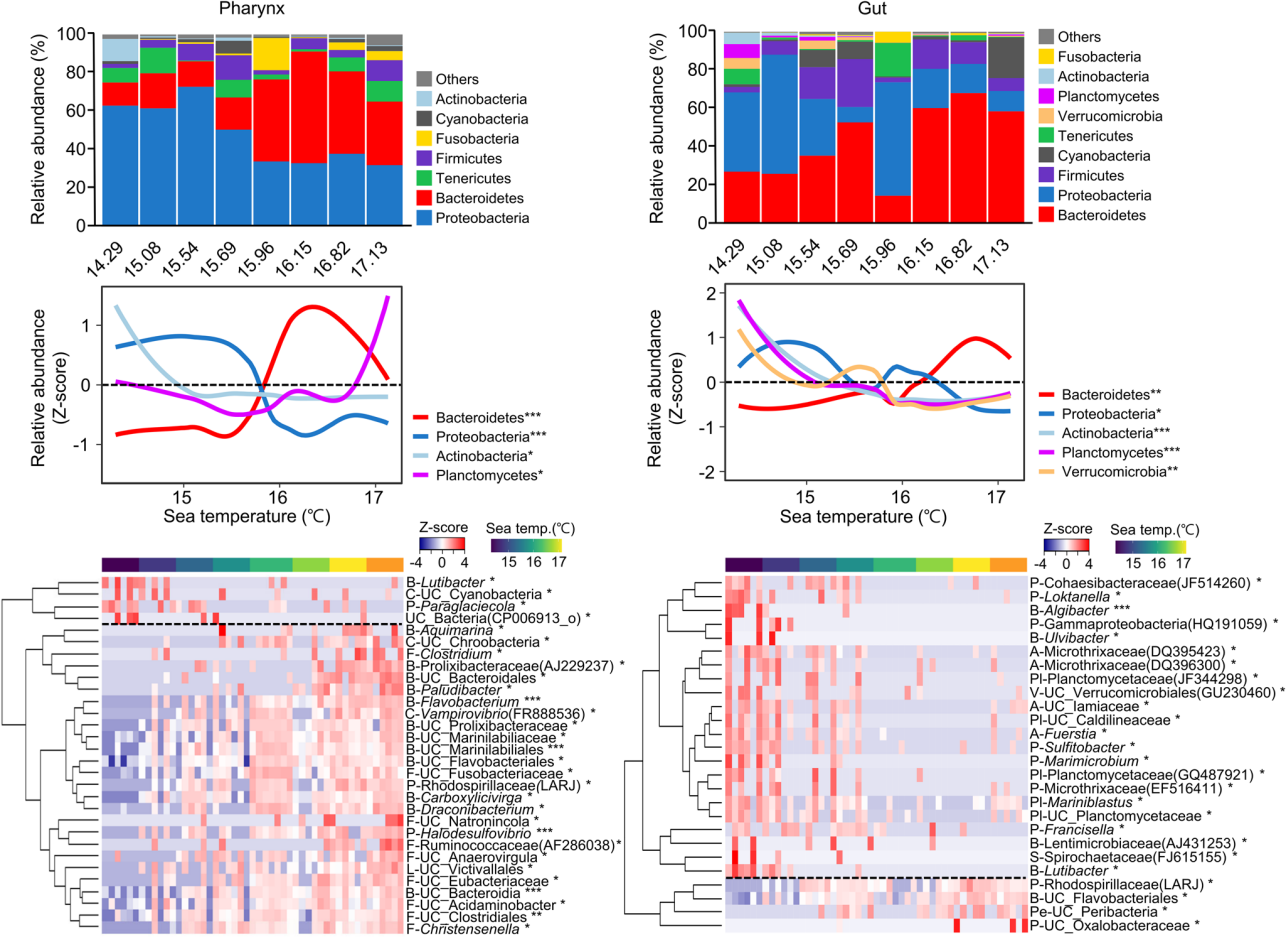
Analysis and comparison of the microbiota and increasing seawater temperature between the pharynx and gut of sea urchins. Bar plots show the difference in microbiota composition according to seawater temperature at the phylum level. Bar plots show the mean relative abundances of phyla in each group. Phyla with relative abundance < 1% in every sample group were combined with the “others”. Smooth line curves show the changes in phyla along with increasing seawater temperature. The shifts of phyla were compared by using the calculation z-score for the relative abundance of each phylum along with seawater temperature. The significance was calculated using the Spearman correlation analysis. Only significantly changed phyla according to seawater temperature were shown in the plot. The heatmap compares significantly changed genera according to seawater temperature between the pharynx and the gut. Color codes for seawater temperature are listed above the heatmap. Clustering was performed using Spearman’s rank correlation. The genera that showed decreasing proportions with an increase in seawater temperature are shown above the black dashed line; the genera with increasing proportions are shown below the dashed line. The significance was calculated after adjusting for sampling site variation using the MaAsLin2. The phylum of each genus is displayed in front of the genus name. P Proteobacteria; B Bacteroidetes; A Actinobacteria; Pl Planctomycetes; V Verrucomicrobia; C Cyanobacteria; L Lentisphaerota; S Spirochaetes; Pe Peregrinibacteria. *p < 0.05, **p < 0.01, ***p < 0.001.

The relative abundance of Proteobacteria and Actinobacteria decreased as the seawater temperature increased, whereas that of Bacteroidetes increased in both the pharynx and gut microbiota (Spearman correlation, *p* < 0.05; Fig. 4). The shift in Planctomycetes according to seawater temperature differed between the pharynx and gut microbiota (*p* < 0.05). Different changes in the microbiota of the pharynx and gut owing to seawater temperature were detailed at the genus level. To identify changes in the relative abundance of microbiota according to seawater temperature, genera that differed from each other substantially according to increasing seawater temperature were selected using the multivariate association with linear models (MaAsLin2) after adjusting for sampling site variation (MaAsLin2, *p* < 0.05). The number of genera that decreased as seawater temperature increased was lower in the pharynx (4 genera) than in the gut (22). In contrast, the number of genera that increased as seawater temperature increased was higher in the pharynx (26) than in the gut microbiota (4).

### Gut microbiota of sea urchins is significantly altered by barren severity

Although seawater temperature influenced the microbiota in sea urchins, the effects differed between the pharynx and gut microbiota. Barren severity was only significantly associated with the variation in gut microbiota in sea urchins (Supplementary Table 3). The influence of algal composition was higher on the gut microbiota than on the pharynx microbiota (Fig. 3c). However, barren severity also influenced regional differences. The correlation between regional differences and microbiota variation (R^2^ ≥ 0.406) was higher than that between barren severity and gut microbiota (R^2^ = 0.223). Therefore, we analyzed the differences in microbiota in sea urchins according to sampling sites within the same mild and severe barren regions (Supplementary Figure 2). The microbiota in sea urchins varied according to the sampling site, even within the same mild and severe barren regions. These variations were also different between the pharynx and gut microbiota. For pharynx microbiota, the highest diversity was detected at site C, whereas the lowest was at site E within mild barren regions (Dunn’s test, *q* < 0.05). The highest number of bacterial amounts was detected at site C, and the lowest was at site D within the mild barren regions. However, the highest diversity was detected at site E within the mild barren regions, and the lowest diversity was detected at site G within the severe barren regions of the gut microbiota. The highest number of bacterial amounts was detected at site B within the mild barren regions, and the lowest was at site D within the mild barren regions. Differences in microbiota due to the sampling site were also detected in the non-multidimensional scale (NMDS) plots of each pharynx and gut microbiota (PERMANOVA test, *p* < 0.001). Coastal characteristics could be a factor in the variation in microbiota between sites. However, diversity and microbiota dissimilarity were also different between sites within the same coastal characteristics (Rias coast: A, B, and C sites; Island: D and E sites; Coastal terrace: F, G, and H sites). The geographical distance between sampling sites could influence the variation in microbiota owing to the high possibility of dispersal according to distance. The correlation between distance and microbiota dissimilarity showed that the pharynx microbiota could be influenced by geographical distance (Spearman correlation, *p* < 0.001) and that the gut microbiota was not influenced by distance (*p* > 0.05).

**Fig. 5:**
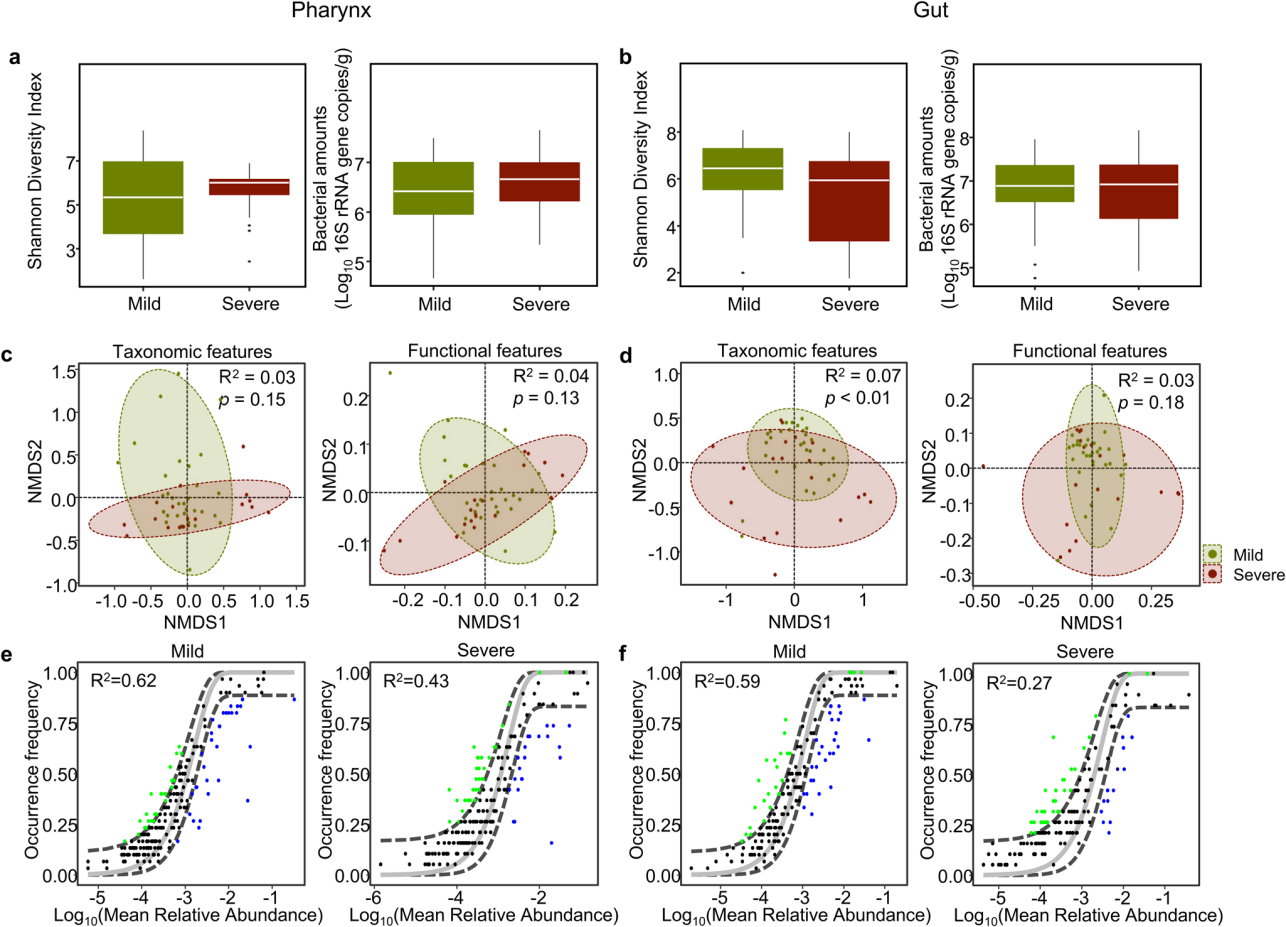
Comparison of the microbiota in the pharynx and gut of sea urchins between mild and severe barren regions. The diversity and bacterial amounts (**a**) in the pharynx and (**b**) in the gut were compared between mild and severe barren regions. Bounds of boxes represent the first quartiles (Q1) and the third quartiles (Q3), center lines represent the median values, and the whiskers stretch to 1.5 times. The significance was calculated using the Wilcoxon-rank sum test. The taxonomic features and predicted functional features of microbiota (**c**) in the pharynx and (**d**) in the gut were compared between mild and severe barren regions in NMDS plots. The *p*-value was calculated using PERMANOVA. Comparison of fit to the neutral model for (**e**) pharynx microbiota and (**f**) gut microbiota between the mild and severe barren regions. The fit to the neutral models was higher in the mild barren region than in the severe barren regions for both the microbiota in the pharynx and gut. The fitness to the model (R^2^) was higher in the pharynx microbiota than in the gut microbiota in both the mild and severe barren regions. Dashed lines represent 95% CIs around the model prediction (gray line). Light green circles indicate above the neutral prediction, blue circles indicate below the prediction, and black circle indicate in the prediction.

The differences in microbiota due to sampling sites could influence microbiota analysis in sea urchins according to barren severity. Therefore, we reduced the differences due to sampling site by using core microbiota from each mild and severe barren region to identify the different features of the core microbiota according to severity. The diversity and bacterial amounts were not significantly different between the mild and severe barren regions in the pharynx and gut microbiota (Wilcoxon-rank sum test, *p* > 0.05; Fig. 5a and b). Although the taxonomic and functional differences in the pharynx microbiota according to barren severity were not significant, the gut microbiota was significantly different according to barren severity (PERMANOVA test, *p* < 0.01; Fig. 5c and d). These results were consistent with the correlation analysis using the EnvFit model (Supplementary Table 3). However, the overall predicted functions of the gut microbiota in the 3rd KEGG category were not significantly different (*p* > 0.05). The neutral model was applied to compare the neutral dispersal of microbes between the microbiota in mild and severe barren regions (Fig. 5e and f). The values of R^2^ were higher in the pharynx microbiota than in the gut microbiota in both the mild and severe barren regions. The fit to the neutral model was higher in the gut microbiota of the mild barren regions (R^2^ = 0.59) than in that of severe barren regions (R^2^ = 0.27). These results indicate that the ecological interactions of microbes occurred more in the gut microbiota and in severe barren regions.

### Potential roles of gut microbiota in sea urchins differed according to barren severity

**Fig. 6:**
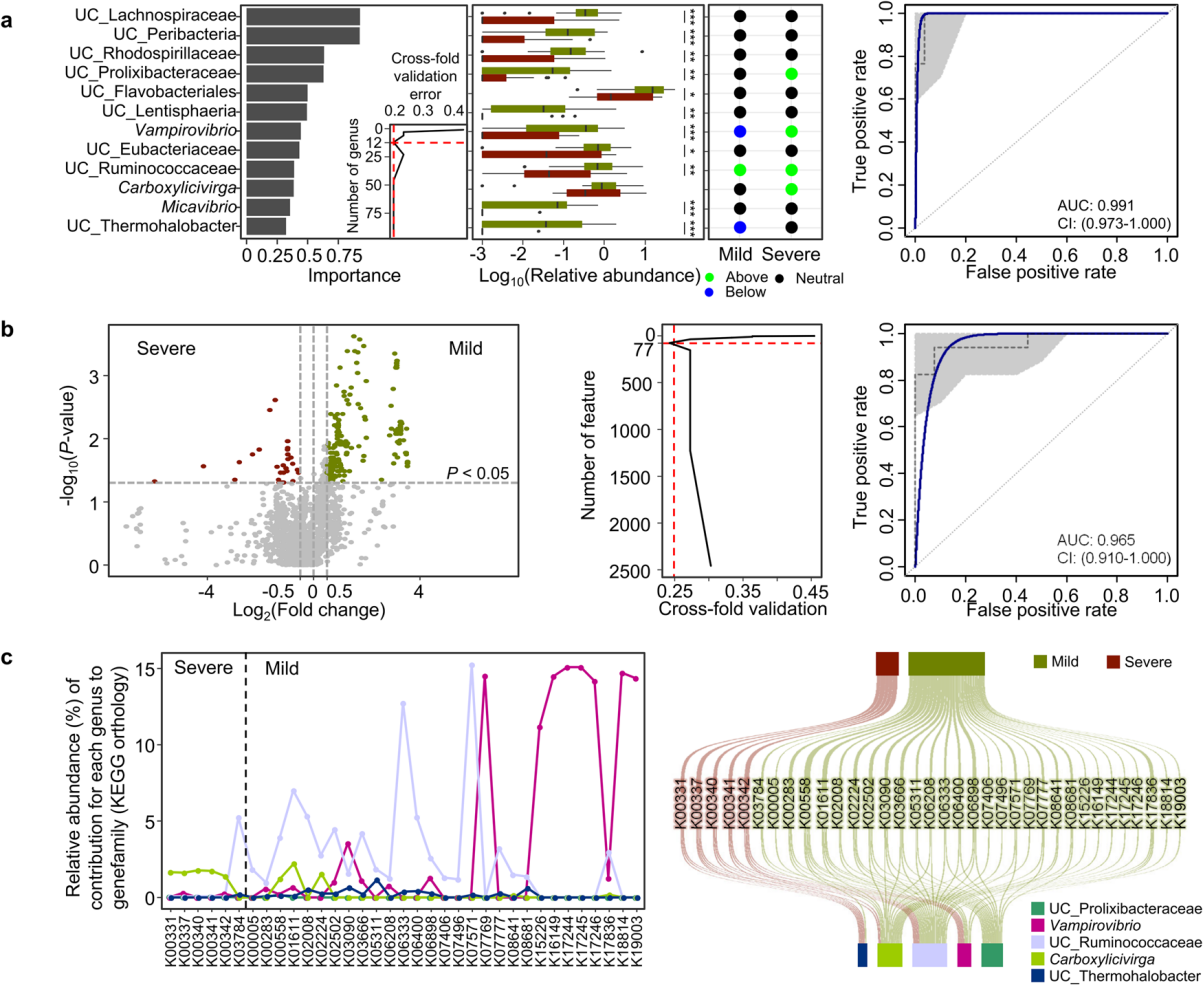
Substantially different genera and functional features in the gut microbiota according to barren severity. **a** Importance of genera in distinguishing the gut microbiota between the mild and severe barren regions. The 12 genera with the most discriminating power were selected using the lowest cross-validation error (inner graph). Relative abundance of selected genera was compared between the mild and severe barren regions in box plots. Bounds of boxes represent the first quartiles (Q1) and the third quartiles (Q3), center lines represent the median values, and the whiskers stretch to 1.5 times. The significance was calculated using the Wilcoxon-rank sum test. *p < 0.05, **p < 0.01, ***p < 0.001. The fit to the neutral models for selected genera was compared between the mild and severe barren regions. An AUROC curve validated the discriminating power of selected genera. The performance during cross-validation and 95% CIs are shown. Overall accuracy was 84.0%. **b** The volcano plot shows the different functional features of gut microbiota between mild and severe barren regions. Functional features with fold changes > |1.5| and *p* < 0.05 (gray dashed line) were considered significant features. The *p*-value was calculated using the Wilcoxon-rank sum test. The lowest cross-validation error selected 77 features with the most discriminating power among significant features. An AUROC curve validated the discriminating power of selected features. The overall accuracy was 80.0%. **c** The contributions of the selected genera to the significant functional features were compared between the mild and severe barren regions. The width of rectangles indicates the frequency of contribution for each genus to gene families in the Sankey diagram.

The severity of urchin barren remarkably influenced the gut microbiota in sea urchins, and the ecological dynamics were predicted to be higher in the gut microbiota than in the pharynx microbiota. Therefore, we further analyzed alterations in the gut microbiota related to barren severity. Twelve genera were selected as significantly different genera in the gut microbiota between the mild and severe barren regions using a random forest model after cross-fold validation (Fig. 6a). The relative abundance of these genera was higher in the gut microbiota of sea urchins obtained from the mild barren regions than in that of the sea urchins obtained from severe barren regions (Wilcoxon-rank sum test, *p* < 0.05, expect for *Carboxylicivirga*). The positions of these genera in the neutral model were compared to predict ecological adaptation in the gut microbiota according to barren severity. *Vampirovibrio* and UC_Ruminococcaceae have commonly adapted genera in the gut microbiota of sea urchins from both mild and severe barren regions. UC_Thermohalobacter was adapted to the mild barren regions, whereas UC_Prolixibacteraceae and *Carboxylicivirga* were adapted to the severe barren regions. The area under the receiver operating characteristics (AUROC) curve indicated that the selected 12 genera could show differences in the gut microbiota in sea urchins between the mild and severe barren regions (AUC = 0.991 and accuracy = 0.84).

A total of 272 predicted gene families from the gut microbiota were significantly different between the mild and severe barren regions (≥1.5 fold-change and Wilcoxon-rank sum test, *p* < 0.05; Fig. 6b). Among these, 77 features were evaluated as different features using the random forest model after cross-fold validation. Differences in these features were evaluated using the AUROC curve (AUC = 0.965 and accuracy = 0.80). The contribution of significantly different genera to the selected gene families was analyzed and compared between the mild and severe barren regions (Fig. 6c). Different genera in the gut microbiota between the mild and severe barren regions significantly contributed to 35 gene families (Wilcoxon-rank sum test, *p* < 0.05). In the gut microbiota of sea urchins from severe barren regions, *Carboxylicivirga* was the main contributor to the gene families involved in oxidative phosphorylation (K00331, K00337, K00340, K00341, and K00342). In contrast, *Vampirovibrio* mainly contributed to the gene families involved in membrane transport, amino acid metabolism, starch and sucrose metabolism, and lipid metabolism, and UC_Ruminococcaceae contributed to the functional features of propanoate metabolism, amino acid metabolism, membrane transport, metabolism of cofactors and vitamins, and biosynthesis of secondary metabolites in the gut microbiota from mild barren regions. These results indicate that the gut microbiota in sea urchins could play different roles depending on the severity of barren.

## Discussion

We analyzed the microbiota in the pharynx and gut of sea urchins according to the severity of the urchin barren. The microbiota in sea urchins was significantly different from that of their habitats, and the microbiota was influenced more by environmental conditions than by host growth status. Regional differences in microbiota were associated with seawater temperature and algal composition at each site. The gut microbiota of sea urchins was strongly influenced by barren severity. These results indicate that the gut microbiota in sea urchins is altered according to the urchin barren, and the microbiota could play different roles in the gut of sea urchins based on the available diet source and barren severity.

In our study, the diversity and bacterial amounts in the gut microbiota in sea urchins were higher than those in the pharynx microbiota. Bacteroidetes was the predominant phylum in the gut microbiota, whereas Proteobacteria was predominant in the pharynx microbiota, similar to the microbiota in their habitats. A higher diversity of gut microbiota than that of the pharynx microbiota was reported in a previous study^31^. The dominance of Bacteroidetes in the gut microbiota was reported in five sea urchin species^33^. Similar microbiota in the pharynx and their surrounding environments could be due to rapid interactions with the external water environment and short retention time of the ingested feed in the esophagus^40^. The gut environment, including lower oxygen concentrations and interactions with digestive enzymes, can differentiate the microbiota of the gut from those of the pharynx and habitats^41^. Bacteroidetes in the gut can play a role in the degradation of proteins and carbohydrates with numerous carbohydrate-active enzymes that can degrade various substrates in plants, algae, and animals^42^. Although a variation in microbiota among the species of sea urchins has been reported^33^, the microbiota shared by sea urchins was also detected in this study.

We applied the neutral model to predict the assembly of microbiota based on the effects of random dispersal from their habitats or ecological dynamics, including microbe-microbe interactions and microbe-host interactions^37^. The higher fit to the neutral assembly model in the pharynx microbiota (R^2^ = 0.51) than in the gut microbiota (R^2^ = 0.45) explained the difference in microbiota between the pharynx and gut. This result also indicated that the possibility of random dispersal from the surrounding environment was higher in the pharynx microbiota than in the gut microbiota. Although Flavobacteriaceae and Ruminococcaceae were previously reported as dominant members of the gut microbiota of sea urchins^31,35,43^, our results indicate that their presence could be a result of neutral processes of drift and dispersal from seawater and sand. However, *Arcobacter*, UC_Hepatoplama, and *Illumatobacter* have been reported as members of the gut microbiota^31,44^, and these bacteria were predicted to be ecologically adapted microbes in the gut. The neutral model used in this study can provide information on the possible influence of the surrounding external microbiota on the microbiota in sea urchins.

Seawater temperature and host growth status could influence the microbiota in sea urchins as well as the dispersal of surrounding microbiota. Although the growth stage was associated with the gut microbiome of *M. nudus* in a previous study^35^, host growth status was associated only with the pharynx microbiota in the present study, and environmental factors were more significant factors in the variation of the microbiota. This difference could be owing to the different experimental designs of the studies. In the previous study, collected sea urchins were cultured in the laboratory, and the microbiome was analyzed during the culturing time. However, our study analyzed the microbiota from collected samples without culture. Seawater temperature affected the pharynx and gut microbiota differently. The diversity of the pharynx microbiota increased with increasing seawater temperature, whereas that of gut microbiota decreased with increasing seawater temperature, as previously reported^45^. However, the bacterial amounts were substantially increased only in the gut. Therefore, some bacteria were more predominant in the gut with increasing seawater temperature. These results also indicate that the pharynx microbiota could have more chances of exposure to diverse microbes in the external microbiota and that the gut microbiota had ecological interactions, such as competition between flourishing and moribund bacteria in the gut, with increasing seawater temperature.

The alteration in microbiota in sea urchins with increasing seawater temperature could be caused by algal composition and optimal growth temperature of bacteria. Several studies have shown the effects of diet composition on the gut microbiota of sea urchins^46,47^. The algal composition differed according to seawater temperature, and the correlation of algal composition with the gut microbiota was higher than that with pharynx microbiota. Therefore, the gut microbiota of sea urchins could be affected by diet composition, which could be related to the severity of urchin barren. Indeed, the effect of barren severity was significant only for the gut microbiota. These results indicate that the gut microbiota in sea urchins is influenced by the available diet source according to barren severity as well as seawater temperature.

The variation in gut microbiota in sea urchins was high, even within the same mild and severe barren regions. These variations were unrelated to the geographical distance between the sampling sites or coastal characteristics. Interpretation of microbiota in natural habitats is difficult because of the influence of complex factors on the microbiota. To distinguish between the influences of barren severity on the gut and pharynx microbiota of sea urchins, we reduced sampling site-specific variations under complex external conditions. Barren severity only influenced the gut microbiota, and the ecological dynamics of gut microbiota and microbiota in severe barren regions were higher than those in the pharynx microbiota and microbiota in mild barren regions. The different genera between the mild and severe barren regions were detected and evaluated using different statistical tools to increase the accuracy of results. The relative abundance of these genera was higher in the gut microbiota of sea urchins from mild barren regions than in those of the sea urchins from severe barren regions. However, the ecological dynamics of these genera in the gut microbiota differed between the mild and severe barren regions. Four genera were above the predicted neutral model in severe barren regions, which indicated that they were adapted to and important in the gut microbiota of severe barren regions despite their low relative abundance. *Carboxylicivirga* is a biomarker in the gut microbiome with pelleted feed, which is made of plant meals lacking algae^44^. Therefore, the adaptation of this genus to the gut microbiota of severely barren regions is consistent with that reported in a previous study. *Vampirovibrio* is an epibiotic parasitic bacterium that attaches to the surface of green algae (*Chlorella*)^48^. The higher abundance of *Vampirovibrio* in mild barren regions than in severe barren regions could be related to the higher amount of feeding algae.

Altered gut microbiota due to barren severity could be related to the function of gut microbiota in sea urchins. Different tools were used to identify and evaluate significantly different functional features of the gut microbiota between mild and severe barren regions. Functional features related to significantly different genera according to barren severity were analyzed in detail. *Vampirovibrio* and UC_Ruminococcaceae mainly contributed to the membrane transport, amino acid metabolism, starch and sugar metabolism, lipid metabolism, and the biosynthesis of secondary metabolites in the gut of sea urchins in the mild barren regions. Microbes in the gut of sea urchins play important roles in carbohydrate, amino acid, lipid, and energy metabolism owing to the lack of certain digestive enzymes in the gut of sea urchins^31,49,50^. Therefore, the gut microbiota in mild barren regions still plays a role in digestion and energy metabolism by feeding on algae. However, *Carboxylicivirga* mainly contributes to oxidative phosphorylation in the gut of sea urchins in severe barren regions. Oxidative phosphorylation is a process of energy metabolism in the mitochondria of eukaryotes and the outer membrane of prokaryotes. Sea urchins consume various algae and sessile invertebrates^51^. Algal food sources for sea urchins are limited in severely barren regions, and the gut microbiota can help to digest recalcitrant materials and transfer nutrients to the urchins^42,52^. These results indicate that the gut microbiota of sea urchins in severely barren regions could play different roles in host energy metabolism with limited feeding algae. Although the effect of diet on the gut microbiota of sea urchins has been reported^35,44^, the influence of urchin barren on the microbiota of sea urchins is limited. Therefore, our results can provide insights into the interactions between gut microbiota and sea urchins under limited dietary conditions of urchin barren in natural habitats. As the predicted functions of microbiota were obtained using PICRUSt2 in this study, these possible functions should be validated. Further studies are necessary to validate and analyze host-microbiome interactions in sea urchins according to barren severity using whole-metagenomics and metabolomics.

In conclusion, sea urchin pharynx and gut microbiota differed according to organ specificity and the possibility of neutral dispersal from the external microbiota. Although environmental factors influence the microbiota in sea urchins, the gut microbiota is substantially altered by the urchin barren. The composition of the gut microbiota and their predicted functions were significantly different between the mild and severe barren regions. The contribution of the gut microbiota to the host metabolism could differ according to the availability of algal feed sources. Although more meta-omics data and experiments are needed to evaluate the results of this study, the influence of urchin barren on the microbiota of sea urchins was determined using several analyses. Our results shed light on the potential role of the microbiota in sea urchins according to the available diet resources. These findings can advance the ecological concept of the dynamic interaction of coexisting organisms in the kelp deforest area.

## Methods

### Sample collection

Sea urchins (*M. nudus*) were collected from five mild barren regions (Taean, Yeosu, Tongyeong, Ulleung do, and Dokdo) and three severe barren regions (Homigot, Guryongpo, and Goseong) between June and July 2021 (Fig. 1). Sampling sites were selected based on the annual report on population dynamics data of sea urchins and the survey report of urchin barren located in the South Korean coast (Supplementary Tables 5, 6, and 7)^53,54^. The barren status was determined based on the cover degree of crustose corallines in the survey region (normal: cover degree < 40%, mild: 40% ≤ cover degree < 80%, and severe: cover degree ≥ 80%) using hyperspectral aerial imaging by the Korea Fisheries Resources Agency (FIRA)^55^. Sea urchins (*n* = 7) were randomly collected from each site and transported to the laboratory in a sterilized container with an ice-chest cooler. We measured the weight, height, and diameter of each sea urchin before separating the pharyngeal and gut tissues (Supplementary Table 1). The pharynx and gut tissues were excised from each sea urchin using the sterile knife, forceps, and scissors. The separated tissues were transferred into sterilized 2-mL tubes and stored at −80 °C before extracting metagenomic DNA.

Sea urchin habitat samples (sand and seawater) were collected from three sites (Ulleung do, Dokdo, and Guryongpo) that were selected according to barren severity. While Ulleung do and Dokdo were the mildest regions, Guryongpo was the most severe region among sampling sites on the eastern coast, which has been maintaining barren regions more consistently than the other South Korean coasts (Supplementary Table 5). Continuous ecological surveys have been conducted in these regions by the Korean Ministry of Ocean and Fisheries and the Korea Fisheries Resource Agency (Supplementary Table 7). As sea urchins were collected from two different sand regions in Guryongpo, two sand samples (surrounding habitat for sea urchins) were collected from this site. Sand (≥ 1 kg) and seawater (2 L) samples were collected in sterilized containers and transported to the laboratory in an ice-chest cooler. Seawater samples were filtered using sterile MF-Millipore membrane filters (50-mm diameter and 0.22-µm pore size; Merck-Millipore, Inc., Burlington, MA, USA) via vacuum filtration. Each membrane filter was transferred into sterilized tubes, and samples were stored at −80 °C before metagenomic DNA extraction.

### DNA extraction and 16 S rRNA amplicon sequencing

Metagenomic DNA was extracted from each sample (pharynx and gut of sea urchin, sand, and seawater filtered membrane) using the RNeasy PowerMicrobiome Kit (Qiagen, Inc., Valencia, CA, USA) and purified using the DNeasy PowerClean Pro Cleanup Kit (Qiagen), according to the manufacturer’s instructions. The V1–V3 region of the 16 S rRNA gene was amplified, based on the protocol for preparing a 16 S metagenomic sequencing library, using the MiSeq system (Illumina, Inc., San Diego, CA, USA), as described previously^56,57^. Briefly, the first amplification was performed with a final volume of 25 µL comprising 1.25 U Ex Taq polymerase (Takara Bio, Shiga, Japan), 2.5 µL of 10× Ex Taq buffer, 4 µL of dNTP mixture, 20 µM of each primer, and 2.5 µL of the template (sample DNA) using a C1000 thermal cycler (BioRad, Hercules, Germany) under the following conditions: initial denaturation at 95 °C for 3 min; 25 cycles of denaturation at 95 °C for 30 s, annealing at 55 °C for 30 s, and extension at 72 °C for 30 s; and final extension at 72 °C for 5 min. Purification and size selection were performed using HiAccuBead (AccuGene, Inc., San Diego, CA, USA). Index polymerase chain reaction (PCR) was performed using the Nextera XT v2 Index Kit (Illumina) and the following program: initial denaturation at 95 °C for 3 min; 8 cycles of denaturation at 95 °C for 30 s, annealing at 55 °C for 30 s, and extension at 72 °C for 30 s; and final extension at 72 °C for 5 min. Purification and size selection were performed using HiAccuBead. Quantifying each amplicon library was performed using a Qubit^TM^ dsDNA HS Assay Kit (Thermo Fisher Scientific, Inc., Waltham, MA, USA). Equimolar concentrations of each library were pooled and sequenced on the Illumina MiSeq system (300-bp paired ends) according to the manufacturer’s instructions. Previous studies have reported that the potential contaminants in low-biomass samples during the experimental process of sequencing-based studies could produce biased results^38,39^. Therefore, negative controls were included at every step to check for contaminants; a total of 27 negative controls were sequenced along with the samples. The negative controls included surface swabs of empty sampling containers, sample stored tubes, a stainless tray (used for cutting off pharynx and gut tissue), seawater filtering tools, a filtering membrane, DNA-free water added to the DNA extraction kit, DNA-free water added to the purification kit, and DNA-free water added to the amplicon library preparation kit.

### Quantitative real-time PCR

As described previously, the relative bacterial amount in each sample was estimated and compared using quantitative real-time PCR based on the 16 S rRNA gene^56,58^. Amplification was performed with a PCR Thermal Cycler Dice Real Time System III (Takara Bio) using the following primers: 340 F (5′-TCCTACGGGAGGCAGCAG-3′) and 518 R (5′-ATTACCGCGGCTGCTGG-3′). For each sample, reactions were performed in triplicate with a final reaction volume of 25 µL, comprising 12.5 µL of 2× TB Green Premix Ex Taq (Tli RNaseH Plus; Takara Bio), 20 µM of each primer, and 2 µL of DNA template (ten-fold diluted metagenomic DNA) or distilled water (negative control). The amplification program was as follows: initial denaturation at 95 °C for 30 s, 40 cycles of denaturation at 95 °C for 5 s and annealing at 60 °C for 30 s, with a final extension at 72 °C for 10 min. Standard curves were generated by performing serial dilution representing log concentrations of the copy number of the 16 S rRNA gene from *Escherichia coli* K12 w3110. The regression coefficient (r^2^) for all standard curves was ≥ 0.99.

### Sequence data processing

Sequences obtained from the MiSeq system were analyzed using the QIIME2 pipeline^59^. Quality filtering, denoising, paired sequence merging, and chimera sequence removal were performed using DADA2 in QIIME2^60^. Amplicon sequence variants (ASVs) from the DADA2 results were assigned taxonomic positions using the BLAST classifier with the ExTaxon-e database^61^. Potential contaminants in the sequencing data were removed using the R package “decontam” based on the sequences of the negative controls^62^. Decontamination was performed with a threshold of 0.5 using prevalence methods in “*isContaminant*” function. Taxonomic and diversity analyses were performed after the decontamination step. Diversity indices were calculated after the rarefication of all samples without replacement.

### Analysis of microbiota

The influences of covariates (host factors: height, weight, and diameter; and environmental factors: sampling site, coast characteristic, seawater temperature, and severity of barren) on microbiota were analyzed using the “*envfit*” function within R package vegan (v.2.5-7). The effect size and significance of each covariate in microbiota variation were determined and compared. Significance was determined using permutational multivariate analysis of variance (PERMANOVA) and the Mantel test.

Functions of microbiota in the pharynx, gut, seawater, and sand were predicted using the phylogenetic investigation of communities by reconstruction of unobserved states (PICRUSt2)^63^. The ASV sequences obtained from QIIME2 were used for this analysis, and the KOs for ASVs were obtained using the PICRUSt2 pipeline. The copy numbers of the KOs were normalized using the cumulative sum scaling method.

We used the MaAsLin2 to adjust for confounding factors, including site differences, coastal characteristics, and severity of barren when performing the correlation analysis between microbiota features and seawater temperature^64^. The analyzed features were selected based on > 30% sample prevalence to increase the relevance of results.

The possibility of dispersal by chance or ecological drift of the genera in the microbiota was assessed using the fit of the Solan Neutral Community Model for Prokaryotes^65^. The observed frequency of genus (the proportion of local communities in which each genus was detected) and its abundance in the metacommunity (the mean relative abundance across all local communities) were used as fit parameters describing the migration rate to the neutral model. The fitting of this parameter to the neutral prediction was calculated using non-linear least-squares fitting in the R package minpack.lm^66^. Binomial proportion 95% confidence intervals (CIs) around the model predictions were calculated using the Wilson score interval in “*binconf*” function in the R package Hmisc^67^.

Comparisons of dominant genera among samples (pharynx, gut, seawater, and sand) and genera significantly correlated with seawater temperature were analyzed using a heatmap plot. The colors of the heatmap correspond to the z-scores for the normalized abundances of each genus among the samples. Dendrogram clusters were computed as a “*hclust*” function with the Spearman correlation, and a heatmap was generated using “*heatmap.2*” function in the R package gplots.

Core features (microbiota and predicted functions) were determined to identify shared features among samples and were compared according to the severity of barren. Core feature analysis was performed using the “*venn*” function within the R package eulerr. The analyzed features were selected based on > 30% sample prevalence to increase the relevance of results.

The random forest model was used to identify different microbiota features of sea urchins between the mild and severe barren regions using the R package, randomForest. We randomly selected 20 sea urchins from the mild barren regions and 13 from the severe barren regions for the training set. To find a robust mtry (number of variables per node in random forest model), algorithm tuning was performed using the “*tuneRF*” function. The classification model was obtained based on a training set and evaluated whether the classification model was suitable for the test. The N top discriminatory features in each dataset were determined by 10-fold cross-validation using the “*rfcv*” function. The AUROC curve was calculated using the “*plot.roc*” function in the R package pROC to examine the accuracy of discriminatory features. 95% CIs was calculated using the “*ci.se*” function with 2000 stratified bootstrap replicates.

A volcano plot was used to visualize the significantly different functional features of the gut microbiota between the mild and severe barren regions. The x-axis shows the fold-change of gene families (log_2_ fold-change) predicted by PICRUSt2, and the y-axis shows significance with -log_10_(*p*-value) calculated using the Wilcoxon rank sum test. The threshold was a fold-change > |1.5| and *p*-value < 0.05. A total of 272 gene families significantly differed in the gut microbiota between mild and severe barren regions.

The contribution of genera to the significantly different gene families is shown using a Sankey diagram. The width of the rectangles represents the frequency of the gene family contribution. A Sankey diagram was generated using the “*ggsankey*” function in the R package ggplots.

### Statistical analysis

The significantly different microbiota features (taxonomic and functional features) between the two groups were determined using the Wilcoxon-rank sum test in the R software. Dunn’s multiple comparison test was used to identify differences between two or more groups using the “dunn.test” R package. *P* values were adjusted using the Benjamini–Hochberg false discovery rate multiple testing correction. Differences in beta-diversity were visualized using NMDS plots based on Bray-Curtis dissimilarity, and significance was determined using PERMANOVA (Adonis from the package vegan with 999 permutations). The correlations of microbiota in sea urchins with seawater temperature, algal composition, and geographical distance were analyzed using the Spearman correlation with “*rcorr*” function in the Hmisc R package. Results with *p* < 0.05 and *q* (adjusted *p* value) < 0.05 were considered statistically significant. All statistical tests were two-sided.

### Reporting summary

Further information on research design is available in the Nature Research Reporting Summary linked to this article.

### Supplementary information

Supplementary Information

Reporting summary

## Data Availability

The sequence reads obtained from this study are available in the EMBL SRA database under the study number PRJEB57350.

## Code availability

Codes used in this study are provided in the supplemental information associated with this manuscript.
