# Supplementary material for: Alterations in sea urchin (Mesocentrotus nudus) microbiota and their potential contributions to host according to barren severity
Source: NPJ Biofilms Microbiomes. 2023 Oct 31;9:83. doi: 10.1038/s41522-023-00450-z (PMC10618176; doi:10.1038/s41522-023-00450-z)
Supplement: Supplementary file 1 — Supplementary Information [file 41522_2023_450_MOESM1_ESM.pdf]

Supplementary Information

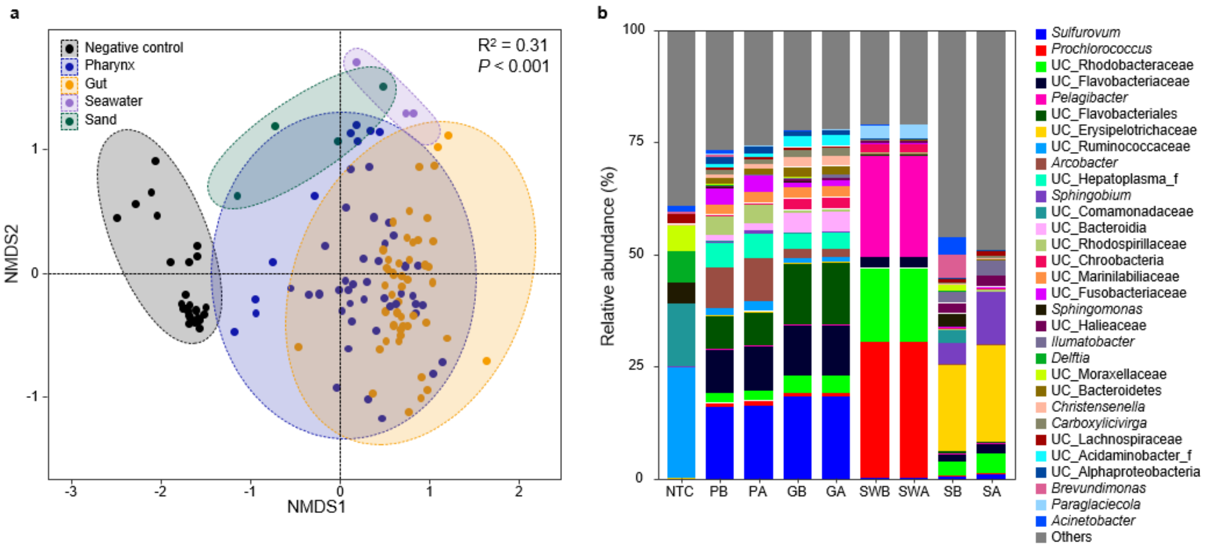

Supplementary Figure 1. Microbiota in samples and negative controls. (**a**) Bacterial compositions in the analyzed samples were compared to composition in negative samples using NMDS plot. Negative controls were surface swabs of empty sampling containers, sample stored tubes, stainless steel tray (for cutting off pharynx and gut tissue), seawater filtering tools, filtering membrane, DNA-free water added to the DNA extraction kit, DNA-free water added to purification kit, and DNA-free water added to the library preparation kit. The *p-value* was calculated using PERMANOVA. (**b**) Changes in genus composition in each sample before and after removal of potential contaminants were compared. Potential contaminants in sequenced data were removed based on detected sequences in negative controls by the Decontam pipeline. A list of trimmed sequences is summarized in Supplementary Table S2. NTC, Negative controls; PB, Pharynx before decontam; PA, Pharynx after decontam; GB, Gut before decontam; GA, Gut after decontam; SWB, Seawater before decontam; SWA, Seawater after decontam; SB, Sand before decontam; SA, Sand after decontam.

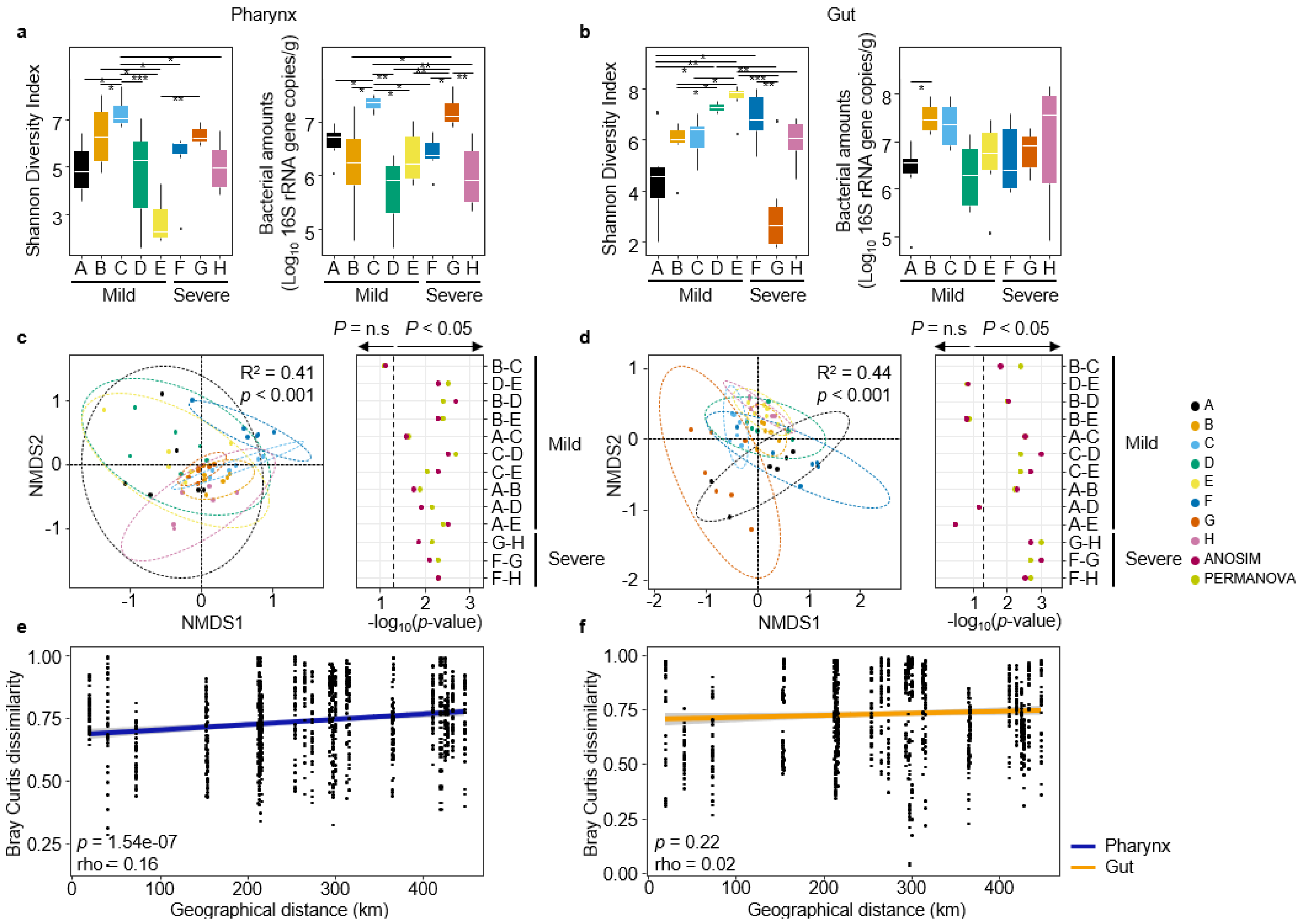

Pharynx
Gut
a
b
c
d
e
f
Shannon Diversity Index
Bacterial amounts (Log10 16S rRNA gene copies/g)
A B C D E F G H
Mild
Severe
NMDS1
NMDS2
R² = 0.41
p < 0.001
R² = 0.44
p < 0.001
P = n.s
P < 0.05
-log10(p-value)
B-C
D-E
B-D
B-E
A-C
C-D
C-E
A-B
A-D
A-E
G-H
F-G
F-H
ANOSIM
PERMANOVA
Bray Curtis dissimilarity
Geographical distance (km)
p = 1.54e-07
rho = 0.16
p = 0.22
rho = 0.02
Pharynx
Gut

Supplementary Figure 2. Variation of microbiota in sea urchin according to sampling sites within mild and severe barren regions. (**a**) The diversity and bacterial amounts in pharynx microbiota were compared among sampling sites. (**b**) The diversity and bacterial amounts in gut microbiota were compared among sampling sites. *$q < 0.05$, **$q < 0.01$, ***$q < 0.001$. (**c**) The comparison of pharynx microbiota among sampling sites in NMDS plot. The significance of difference between sampling sites were calculated by using ANOSIM and PERMANOVA. The difference of microbiota between sampling sites were compared by *p* value. (**d**) The comparison of gut microbiota among sampling sites in NMDS plot. The significance of difference between sampling sites were calculated by using ANOSIM and PERMANOVA. The difference of microbiota between sampling sites were compared by *p* value. A, Taean; B, Tongyeong; C, Yeosoo; D, Ulleng do; E, Dokdo; F, Goseong; G, Homigot; H, Guryongpo. (**e**) Correlation between pharynx microbiota and geographical distance was analyzed using the Spearman rank correlation. (**f**) Correlation between gut microbiota and geographical distance was analyzed.

Supplementary Table 1. Characteristics of sampling site and collected sea urchins in each site

	Characteristic	Sampling site							
		A	B	C	D	E	F	G	H
Habitat	Site	Taean	Tongyoung	Yesoo	Ulleng do	Dokdo	Gosung	Homigot	Gooryongpo
	Coast characteristic	Rias coast	Rias coast	Rias coast	Island	Island	Coastal terrace	Coastal terrace	Coastal terrace
	Seawater Temperature (°C)	15.08	17.13	16.82	15.54	15.69	14.29	15.96	16.15
	pH	N.D[b]	8.12	8.01	N.D	N.D	8.22	8.22	8.21
	Salinity (PSU[a])	N.D	33.3	32	32.8	32.6	32.9	32.7	32.8
	Barren severity	Mild	Mild	Mild	Mild	Mild	Severe	Severe	Severe
	Normal area (%)	90.21	84.2	84.9	81.9	81.9	53.1	40.7	40.7
	Mild area (%)	3.54	8.5	8.9	11.9	11.9	31.4	34.2	34.2
	Severe area (%)	6.25	7.3	6.2	6.2	6.2	15.5	25.1	25.1
The number of samples (n)	Randomly collected sea urchin	7	7	7	7	7	7	7	7
	Analyzed sea urchin	6	6	6	6	6	6	7	6
	Seawater (n)	0	0	0	1	1	0	0	1
	Sand (n)	0	0	0	1	1	0	0	2
Sea urchin (mean ± standard deviation)	Weight (g)	123.32 ± 30.14	72.83 ± 22.06	77.04 ± 19.53	112.84 ± 25.85	56.55 ± 43.03	40.67 ± 9.97	50.91 ± 7.43	39.71 ± 15.18
	Diameter (cm)	7.22 ± 1.02	6.00 ± 0.56	6.06 ± 0.57	6.53 ± 0.76	4.24 ± 1.33	4.58 ± 0.49	5.29 ± 0.71	4.98 ± 1.2
	Height (cm)	3.62 ± 0.62	2.77 ± 0.32	3.03 ± 0.4	3.63 ± 0.61	2.11 ± 0.76	2.26 ± 0.33	2.69 ± 0.39	2.53 ± 0.48

[a]PSU: practical salinity unit (g/kg)

[b]N.D: Not determined

Supplementary Table 2. List of trimmed amplicon sequence variants (ASVs) from sequence data of samples determined by the Decontam pipeline

Phylum	Class	Order	Family	Genus	*p*-value	Contaminant
Proteobacteria	Betaproteobacteria	Burkholderiales	Comamonadaceae	__	2.72E-08	TRUE
Proteobacteria	Alphaproteobacteria	Rhizobiales	Phyllobacteriaceae	*Mesorhizobium*	2.64E-09	TRUE
Proteobacteria	Betaproteobacteria	Burkholderiales	Comamonadaceae	*Delftia*	2.59E-22	TRUE
Proteobacteria	Gammaproteobacteria	Pseudomonadales	Moraxellaceae	__	0.0003621	TRUE
Firmicutes	Erysipelotrichi	Erysipelotrichales	Erysipelotrichaceae	*Clostridium_g6*	1.72E-08	TRUE
Proteobacteria	Alphaproteobacteria	Rhodospirillales	Acetobacteraceae	*Roseomonas*	0.032034	TRUE
Actinobacteria	Actinobacteria_c	Bifidobacteriales	Bifidobacteriaceae	*Bifidobacterium*	1.72E-08	TRUE
Actinobacteria	Actinobacteria_c	Propionibacteriales	Propionibacteriaceae	*Cutibacterium*	1.48E-07	TRUE
Firmicutes	Clostridia	Clostridiales	Ruminococcaceae	*Monoglobus*	3.00E-10	TRUE
Proteobacteria	Alphaproteobacteria	Caulobacterales	Caulobacteraceae	*Brevundimonas*	0.5	TRUE
Firmicutes	Clostridia	Clostridiales	Lachnospiraceae	*Blautia*	3.54E-08	TRUE
Proteobacteria	Alphaproteobacteria	Sphingomonadales	Sphingomonadaceae	*Sphingomonas*	6.02E-09	TRUE
Proteobacteria	Alphaproteobacteria	Caulobacterales	Caulobacteraceae	__	3.72E-23	TRUE
Bacteroidetes	Flavobacteria	Flavobacteriales	Flavobacteriaceae	*Chryseobacterium*	0.0169039	TRUE
Proteobacteria	Gammaproteobacteria	Pseudomonadales	Moraxellaceae	*Acinetobacter*	5.38E-06	TRUE
Bacteroidetes	Bacteroidia	Bacteroidales	Rikenellaceae	*Alistipes*	3.51E-08	TRUE
Proteobacteria	Gammaproteobacteria	Pseudomonadales	Pseudomonadaceae	*Pseudomonas*	0.1623655	TRUE
Firmicutes	Clostridia	Clostridiales	Mogibacterium_f	PAC001609_g	4.28E-13	TRUE
Proteobacteria	Betaproteobacteria	Burkholderiales	Burkholderiaceae	*Burkholderia*	3.49E-16	TRUE
Proteobacteria	Alphaproteobacteria	Sphingomonadales	Sphingomonadaceae	*Novosphingobium*	6.58E-10	TRUE
Firmicutes	Clostridia	Clostridiales	Ruminococcaceae	*Ruminococcus*	0.004841	TRUE
Acidobacteria	Solibacteres	Solibacterales	Solibacteraceae	__	8.76E-10	TRUE
Firmicutes	Clostridia	Clostridiales	Ruminococcaceae	*Ruminococcus_g2*	1.10E-07	TRUE
Firmicutes	Clostridia	Clostridiales	Clostridiaceae	*Arthromitus*	3.77E-06	TRUE

Proteobacteria	Alphaproteobacteria	Rhizobiales	Bradyrhizobiaceae	*Bradyrhizobium*	2.13E-14	TRUE
Actinobacteria	Coriobacteriia	Coriobacteriales	Coriobacteriaceae	__	9.72E-17	TRUE
Actinobacteria	Actinobacteria_c	Corynebacteriales	Corynebacteriaceae	*Corynebacterium*	9.70E-11	TRUE
Actinobacteria	Coriobacteriia	Coriobacteriales	Coriobacteriaceae	*Collinsella*	4.28E-13	TRUE
Firmicutes	Clostridia	Clostridiales	Mogibacterium_f	PAC001168_g	6.72E-09	TRUE
Firmicutes	Clostridia	Clostridiales	Lachnospiraceae	*Ruminococcus_g4*	2.58E-10	TRUE
Firmicutes	Clostridia	Clostridiales	Ruminococcaceae	*Caproiciproducens*	0.0123521	TRUE
Verrucomicrobia	Verrucomicrobiae	Verrucomicrobiales	Akkermansiaceae	*Akkermansia*	2.46E-16	TRUE
Actinobacteria	Actinobacteria_c	Micrococcales	Micrococcaceae	*Kocuria*	0.0141617	TRUE
Firmicutes	Clostridia	Clostridiales	Ruminococcaceae	*Pseudoflavonifractor*	5.36E-08	TRUE
Proteobacteria	Alphaproteobacteria	Rhizobiales	Bosea_f	*Bosea*	0.0004907	TRUE
Proteobacteria	Alphaproteobacteria	Rhizobiales	Hyphomicrobiaceae	*Pedomicrobium*	4.97E-11	TRUE
Actinobacteria	Actinobacteria_c	Corynebacteriales	Mycobacteriaceae	*Mycobacterium*	0.0512175	TRUE
Proteobacteria	Gammaproteobacteria	Steroidobacter_o	Steroidobacter_f	*Acidibacter*	2.84E-15	TRUE
Firmicutes	Clostridia	Clostridiales	Lachnospiraceae	*Anaerotignum*	7.91E-12	TRUE
Cyanobacteria	PAC000053_c	PAC000053_o	PAC000053_f	PAC000053_g	4.55E-06	TRUE
Actinobacteria	Actinobacteria_c	Corynebacteriales	Nocardiaceae	*Gordonia*	1.39E-05	TRUE
Actinobacteria	Actinobacteria_c	Micrococcales	Micrococcaceae	*Micrococcus*	0.0029583	TRUE
Bacteroidetes	Bacteroidia	Bacteroidales	Bacteroidaceae	*Bacteroides*	1.65E-08	TRUE
Firmicutes	Erysipelotrichi	Erysipelotrichales	Erysipelotrichaceae	*Coprobacillus*	0.0001105	TRUE
Firmicutes	Clostridia	Clostridiales	Peptostreptococcaceae	__	0.0028747	TRUE
Actinobacteria	Actinobacteria_c	Propionibacteriales	Nocardioidaceae	*Nocardioides*	0.490745	TRUE
Actinobacteria	Actinobacteria_c	Micrococcales	Microbacteriaceae	*Microbacterium*	3.99E-05	TRUE
Proteobacteria	Betaproteobacteria	Burkholderiales	Comamonadaceae	*Acidovorax*	0.0310976	TRUE
Actinobacteria	Actinobacteria_c	Frankiales	Nakamurellaceae	__	0.0202984	TRUE
Proteobacteria	Alphaproteobacteria	Rhizobiales	Methylobacteriaceae	*Methylobacterium*	1.82E-06	TRUE
Firmicutes	Clostridia	Clostridiales	Lachnospiraceae	*Anaerostipes*	9.24E-08	TRUE

Firmicutes	Clostridia	Clostridiales	Ruminococcaceae	*Eubacterium_g8*	2.20E-05	TRUE
Firmicutes	Clostridia	Clostridiales	Ruminococcaceae	*Sporobacter*	0.0319716	TRUE
Firmicutes	Clostridia	Clostridiales	Ruminococcaceae	*Faecalibacterium*	2.88E-11	TRUE
Firmicutes	Tissierellia	Tissierellales	Peptoniphilaceae	__	7.00E-07	TRUE
Firmicutes	Clostridia	Clostridiales	Christensenellaceae	PAC001207_g	2.07E-07	TRUE
Bacteroidetes	Bacteroidia	Bacteroidales	Porphyromonadaceae	*Parabacteroides*	1.07E-09	TRUE
Proteobacteria	Alphaproteobacteria	Rhizobiales	Rhizobiaceae	*Rhizobium*	0.1642096	TRUE
Proteobacteria	Alphaproteobacteria	Rhizobiales	Xanthobacteraceae	*Xanthobacter*	0.032034	TRUE
Actinobacteria	Actinobacteria_c	Corynebacteriales	Nocardiaceae	*Rhodococcus*	0.0232317	TRUE
Proteobacteria	Gammaproteobacteria	Enterobacterales	Enterobacteriaceae	__	4.23E-08	TRUE
Firmicutes	Clostridia	Clostridiales	Ruminococcaceae	*Oscillibacter*	2.15E-09	TRUE
Proteobacteria	Alphaproteobacteria	Sphingomonadales	Erythrobacteraceae	*Porphyrobacter*	0.4791992	TRUE
Firmicutes	Clostridia	Clostridiales	Lachnospiraceae	*Eubacterium_g5*	3.77E-06	TRUE
Actinobacteria	Actinobacteria_c	Micrococcales	Micrococcaceae	*Rothia*	0.0912491	TRUE
Actinobacteria	Actinobacteria_c	Corynebacteriales	Dietziaceae	*Dietzia*	0.2651413	TRUE
Firmicutes	Clostridia	Clostridiales	Lachnospiraceae	*Clostridium_g24*	2.68E-06	TRUE
Proteobacteria	Betaproteobacteria	Burkholderiales	Comamonadaceae	*Variovorax*	0.0007262	TRUE
Firmicutes	Clostridia	Clostridiales	Lachnospiraceae	*Syntrophococcus*	0.0007412	TRUE
Proteobacteria	Betaproteobacteria	Burkholderiales	Comamonadaceae	*Comamonas*	0.0833076	TRUE
Firmicutes	Clostridia	Clostridiales	Ruminococcaceae	*Eubacterium_g23*	1.82E-06	TRUE
Actinobacteria	Actinobacteria_c	Micrococcales	Dermacoccaceae	__	0.0569917	TRUE
Firmicutes	Clostridia	Clostridiales	Christensenellaceae	PAC001115_g	0.0348792	TRUE
Proteobacteria	Alphaproteobacteria	Sphingomonadales	__	__	0.2045455	TRUE
Firmicutes	Erysipelotrichi	Erysipelotrichales	Erysipelotrichaceae	*Longicatena*	0.0001305	TRUE
Proteobacteria	Betaproteobacteria	Methylophilales	Methylophilaceae	*Methylophilus*	0.032034	TRUE
Proteobacteria	Gammaproteobacteria	Enterobacterales	Enterobacteriaceae	*Escherichia*	0.0007262	TRUE
Firmicutes	Clostridia	Clostridiales	Lachnospiraceae	*Agathobacter*	0.0039035	TRUE

Firmicutes	Tissierellia	Tissierellales	Peptoniphilaceae	*Peptoniphilus*	0.0578495	TRUE
Proteobacteria	Gammaproteobacteria	Enterobacterales	Enterobacteriaceae	*Klebsiella*	8.08E-08	TRUE
Firmicutes	Clostridia	Clostridiales	Lachnospiraceae	*Clostridium_g21*	0.0008333	TRUE
Firmicutes	Clostridia	Clostridiales	Ruminococcaceae	*Ruthenibacterium*	4.28E-13	TRUE
Firmicutes	Clostridia	Clostridiales	Lachnospiraceae	*Clostridium_g34*	2.65E-05	TRUE
Firmicutes	Clostridia	Clostridiales	Lachnospiraceae	PAC001201_g	0.0086577	TRUE
Actinobacteria	Actinobacteria_c	Micrococcales	Micrococcaceae	*Nesterenkonia*	0.0202984	TRUE
Bacteroidetes	Sphingobacteriia	Sphingobacteriales	Sphingobacteriaceae	*Pedobacter*	0.2045455	TRUE
Firmicutes	Tissierellia	Tissierellales	Peptoniphilaceae	*Anaerococcus*	0.0032397	TRUE
Firmicutes	Clostridia	Clostridiales	Ruminococcaceae	*Subdoligranulum*	0.0003072	TRUE
Firmicutes	Bacilli	Lactobacillales	Streptococcaceae	*Streptococcus*	0.032034	TRUE
Firmicutes	Clostridia	Clostridiales	Lachnospiraceae	LLKB_g	0.3029146	TRUE
Armatimonadetes	Fimbriimonadia	Fimbriimonadales	Fimbriimonadaceae	*Fimbriimonas*	0.3029146	TRUE
Chloroflexi	Anaerolineae	PAC000395_o	FJ481317_f	__	0.3029146	TRUE
Firmicutes	Clostridia	Clostridiales	Mogibacterium_f	__	0.0283824	TRUE
Actinobacteria	Actinobacteria_c	Frankiales	Geodermatophilaceae	*Blastococcus*	0.0202984	TRUE
Firmicutes	Clostridia	Clostridiales	Lachnospiraceae	*Acetatifactor*	0.0141617	TRUE
Actinobacteria	Actinobacteria_c	Streptomycetales	Streptomycetaceae	*Streptomyces*	0.2045455	TRUE
Bacteroidetes	Bacteroidia	Bacteroidales	Odoribacteraceae	*Butyricimonas*	0.0202984	TRUE
Proteobacteria	Alphaproteobacteria	Rhodobacterales	Rhodobacteraceae	*Rubellimicrobium*	0.2045455	TRUE
Firmicutes	Clostridia	Clostridiales	Lachnospiraceae	*Lachnospira*	0.0004907	TRUE
Firmicutes	Bacilli	Lactobacillales	Enterococcaceae	*Enterococcus*	0.0001786	TRUE
Synergistetes	Synergistia	Synergistales	Synergistaceae	__	0.4791992	TRUE
Bacteroidetes	Bacteroidia	Bacteroidales	Odoribacteraceae	*Odoribacter*	0.3029146	TRUE
Proteobacteria	Betaproteobacteria	Burkholderiales	Alcaligenaceae	*Achromobacter*	0.0202984	TRUE
Firmicutes	Clostridia	Clostridiales	Lachnospiraceae	PAC000692_g	0.0569917	TRUE
Firmicutes	Clostridia	Clostridiales	Ruminococcaceae	*Agathobaculum*	0.0004907	TRUE

Proteobacteria	Betaproteobacteria	Burkholderiales	Comamonadaceae	*Curvibacter*	0.3029146	TRUE
Firmicutes	Clostridia	Clostridiales	Ruminococcaceae	PAC001144_g	0.0141617	TRUE
Firmicutes	Clostridia	Clostridiales	Christensenellaceae	PAC001360_g	0.1642096	TRUE
Bacteroidetes	Flavobacteria	Flavobacteriales	Flavobacteriaceae	*Cloacibacterium*	0.0283824	TRUE
Firmicutes	Clostridia	Clostridiales	Lachnospiraceae	*Frisingicoccus*	0.0569917	TRUE
Firmicutes	Bacilli	Lactobacillales	Lactobacillaceae	*Lactobacillus*	0.032034	TRUE
Firmicutes	Clostridia	Clostridiales	Lachnospiraceae	KE159571_g	0.0179695	TRUE
Actinobacteria	Actinobacteria_c	Pseudonocardiales	Pseudonocardiaceae	*Pseudonocardia*	0.0202984	TRUE
Firmicutes	Clostridia	Clostridiales	Lachnospiraceae	PAC001200_g	0.0202984	TRUE
Firmicutes	Clostridia	Clostridiales	Ruminococcaceae	*Paludicola*	0.0202984	TRUE
Firmicutes	Negativicutes	Acidaminococcales	Acidaminococcaceae	*Acidaminococcus*	0.2045455	TRUE
Firmicutes	Clostridia	Clostridiales	Ruminococcaceae	PAC000661_g	0.0004334	TRUE
Actinobacteria	Coriobacteriia	Coriobacteriales	Coriobacteriaceae	*Enterorhabdus*	0.0039035	TRUE
Proteobacteria	Alphaproteobacteria	Rhizobiales	Methylobacteriaceae	*Microvirga*	0.0202984	TRUE
Actinobacteria	Actinobacteria_c	Micrococcales	Micrococcaceae	__	0.2943662	TRUE
Firmicutes	Bacilli	Bacillales	Staphylococcaceae	*Staphylococcus*	0.032034	TRUE
Proteobacteria	Gammaproteobacteria	Enterobacterales	__	__	0.3949292	TRUE
Firmicutes	Clostridia	Clostridiales	Lachnospiraceae	*Eubacterium_g17*	0.0141617	TRUE
Bacteroidetes	Bacteroidia	Bacteroidales	Barnesiellaceae	__	0.0202984	TRUE
Actinobacteria	Actinobacteria_c	Actinomycetales	Actinomycetaceae	*Actinomyces*	0.0509961	TRUE
Deinococcus-Thermus	Deinococci	Deinococcales	Deinococcaceae	*Deinococcus*	0.490745	TRUE
Acidobacteria	Blastocatellia	Blastocatellales	Blastocatellaceae	__	0.2045455	TRUE
Firmicutes	Clostridia	Clostridiales	Ruminococcaceae	PAC001402_g	0.2045455	TRUE
Proteobacteria	Alphaproteobacteria	Rhodospirillales	AY957891_f	AY957891_g	0.2045455	TRUE
Actinobacteria	Actinobacteria_c	Propionibacteriales	Propionibacteriaceae	__	0.1313979	TRUE
Firmicutes	Clostridia	Clostridiales	Lachnospiraceae	KE159600_g	0.0569917	TRUE
Bacteroidetes	Bacteroidia	Bacteroidales	AC160630_f	DQ677001_g	0.0569917	TRUE

Proteobacteria	Alphaproteobacteria	Rhodospirillales	Acetobacteraceae	*Craurococcus*	0.2045455	TRUE
Firmicutes	Clostridia	Clostridiales	Lachnospiraceae	PAC000664_g	0.0569917	TRUE
Actinobacteria	Actinobacteria_c	Micrococcales	Micrococcaceae	*Glutamicibacter*	0.3029146	TRUE
Firmicutes	Clostridia	Clostridiales	Lachnospiraceae	PAC001092_g	0.2045455	TRUE
Firmicutes	Clostridia	Clostridiales	Lachnospiraceae	KE159538_g	0.0032397	TRUE
Proteobacteria	Betaproteobacteria	Burkholderiales	Ralstonia_f	*Cupriavidus*	0.3029146	TRUE
Proteobacteria	Gammaproteobacteria	Xanthomonadales	Xanthomonadaceae	__	0.3029146	TRUE
Planctomycetes	Planctomycetia	Planctomycetales	Gemmataceae	*Gemmata*	0.0202984	TRUE
Bacteroidetes	Sphingobacteriia	Sphingobacteriales	Chitinophagaceae	*Sediminibacterium*	0.3949292	TRUE
Firmicutes	Clostridia	Clostridiales	Lachnospiraceae	*Eubacterium_g6*	0.2045455	TRUE
Chloroflexi	Thermomicrobia	DQ129389_o	DQ129389_f	DQ129389_g	0.2045455	TRUE
Firmicutes	Clostridia	Clostridiales	Mogibacterium_f	*Emergencia*	0.2045455	TRUE
Firmicutes	Bacilli	Lactobacillales	Carnobacteriaceae	__	0.2045455	TRUE
Planctomycetes	Planctomycetia	Planctomycetales	Isosphaeraceae	AY234711_g	0.2045455	TRUE

Supplementary Table 3. Correlation between microbiota variation and influencing factors. The correlations were determined using the EnvFit model based on Bray-Curtis dissimilarity of microbiota

Variables	Microbiota in pharynx		Microbiota in gut	
	R-squared	P value	R-squared	P value
Environmental factors				
Region (sampling site)	0.406	0.001	0.443	0.001
Coast characteristic	0.090	0.002	0.139	0.002
Seawater temperature (℃)	0.232	0.001	0.206	0.003
pH	0.058	0.780	0.059	0.203
Salinity (PSU)	0.022	0.595	0.021	0.372
Barren severity (mild vs. severe)	0.064	0.071	0.223	0.001
Host factors				
Weight (g)	0.137	0.021	0.086	0.932
Diameter (cm)	0.093	0.046	0.078	0.970
Height (cm)	0.037	0.307	0.073	0.868

Supplementary Table 4. Biomass ratio of algal composition in each site

Site	Region	Biomass percentage (%)			References
		Chlorophyta	Phaenophyta	Rhdophyta	
A	Taean	4.02	13.90	82.08	S1
B	Tongyoung	7.89	63.23	28.88	S2
C	Yesoo	0.10	93.01	6.89	S2,3
D	Ulleng do	1.45	91.84	6.71	S4,5
E	Dokdo	0.18	97.88	1.95	S6
F	Gosung	1.95	82.19	15.87	S7
G, H	Homigot, Gooryongpo	11.10	64.30	24.60	S7,8

Supplementary Table 5. Summary of the survey report of urchin barren located in the South Korean coast. The survey data was provided by the Korea Fisheries Resources Agency (FIRA; https://www.fira.or.kr). The survey was conducted in 2019 for the eastern and southern coast, and it was conducted in 2020 for the western coast

Coast	Site	Survey area (ha)	Barren area					
			Area (ha)			Ratio (%)		
			Normal	Mild	Severe	Normal	Mild	Severe
Eastern	**Gosung**	358.1	190.4	112.3	55.4	53.2	31.4	15.5
	Yangyang	468.1	270.1	112.0	86.1	57.7	23.9	18.4
	Gangneung	853.0	514.2	190.2	148.6	60.3	22.3	17.4
	Donghae	638.4	369.8	180.1	88.5	57.9	28.2	13.9
	Samcheok	652.2	404.2	153.3	94.7	62.0	23.5	14.5
	**Ulleung do, Dokdo**	794.7	650.6	94.6	49.5	81.9	11.9	6.2
	Uljin	1,004.2	494.9	248.5	260.8	49.3	24.7	26.0
	Yeongdeok	789.3	378.4	182.9	228.0	47.9	23.2	28.9
	**Homigot, Guryongpo**	4,263.1	1,734.0	1,457.0	1,072.1	40.7	34.2	25.1
	Gyeongju	773.2	414.6	207.2	151.4	53.6	26.8	19.6
	Ulsan	1,686.7	825.9	446.0	414.8	49.0	26.4	24.6
	Busan	1,144.0	695.2	279.3	169.5	60.8	24.4	14.8
Southern	Changwon	112.0	106.7	10.9	4.4	87.5	8.9	3.6
	Geoje	726.6	628.3	63.7	34.6	86.5	8.8	4.7
	**Tongyeong**	1,203.4	1,012.9	101.9	88.6	84.2	8.5	7.3
	Sacheon	165.3	155.9	6.0	3.5	94.3	3.6	2.1
	Namhae	558.0	458.9	85.0	14.1	82.2	15.2	2.6
	Hadong	47.3	43.3	3.5	0.5	91.5	7.4	1.1
	**Yeosoo**	1,948.3	1,654.9	173.3	120.1	84.9	8.9	6.2
	Suncheon	1.2	1.2	0.0	0.0	100.0	0.0	0.0
	Gwangyang	0.5	0.5	0.0	0.0	100.0	0.0	0.0
	Boseong	31.7	29.3	2.2	0.2	92.4	6.9	0.7
	Goheung	915.6	861.0	36.1	18.5	94.0	3.9	2.1
	Jangheung	44.6	40.9	1.7	2.0	91.7	3.8	4.5
	Gangjin	11.4	10.4	0.8	0.2	91.2	7.0	1.8
	Wando	2,290.1	2,066.4	141.6	82.1	90.2	6.2	3.6
	Haenam	179.9	161.5	11.6	6.9	89.8	6.4	3.8
Western	Jawoldo	2.2	2.1	0.1	0.1	95.0	2.5	2.5
	Deokjeokdo	3.7	3.5	0.1	0.0	97.2	1.5	1.3

**Taean**	37.4	33.7	1.3	2.3	90.2	3.5	6.3
Gauido	36.1	35.2	0.5	0.4	97.4	1.5	1.1
Baekado	11.0	10.1	0.5	0.3	92.4	4.7	2.9
Seonyudo	14.3	12.4	1.3	0.7	86.6	8.8	4.6
Sapsido	25.6	24.1	0.6	0.9	94.1	2.5	3.4
Hodo	12.8	12.7	0.0	0.1	99.3	0.2	0.5
Eocheongdo	9.5	9.4	0.1	0.0	98.9	0.7	0.4
Oeyeondo	14.5	14.5	0.0	0.0	99.6	0.1	0.3
Nogdo	5.4	4.7	0.4	0.2	88.0	8.0	4.0
Gwanlido	6.4	6.0	0.1	0.2	94.3	2.0	3.7
Byeonsanbando	15.6	14.9	0.4	0.4	95.2	2.3	2.5
Wido	11.3	10.5	0.4	0.3	93.7	3.3	3.1
Myeongdo	12.4	11.4	0.5	0.5	92.0	4.3	3.7
Anmado	19.5	18.5	0.3	0.7	94.7	1.5	3.8
Songido	9.2	8.8	0.4	0.1	94.8	3.9	1.3
Seogeochado	5.6	4.6	0.5	0.6	81.3	8.6	10.1
Bigeumdo	12.2	11.2	0.4	0.7	91.1	3.6	5.3
Dochodo	9.3	8.5	0.6	0.2	90.7	6.7	2.7
Sangjodo	8.7	8.0	0.4	0.3	91.9	4.5	3.6
Hajodo	7.3	6.7	0.2	0.3	92.5	3.0	4.5
Heuksando	9.6	8.5	0.7	0.5	88.2	6.8	5.1
Hongdo	12.7	11.8	0.4	0.5	93.1	3.1	3.8
Gageodo	5.7	5.2	0.2	0.2	92.2	3.8	4.0

Supplementary Table 6. Summary of the annual report on population dynamics data of sea urchins in Republic of Korea. The annual report was based on data from the Korean Statistical Information Service (https://kosis.kr/statHtml/statHtml.do?orgId=101&tblId=DT_1EW0004&conn_path=I3)

Administrative Regions	Sampling Site	Annual reported production of Sea urchins (tons/year)					
		2015	2016	2017	2018	2019	2020
Busan	-	219	203	231	217	313	326
Incheon	-	-	-	-	< 1	-	< 1
Ulsan	-	180	151	108	118	99	234
Gyeonggi-do	-	-	< 1	-	-	-	-
Gangwon-do	Gosung	399	306	249	1,032	892	53
Chungcheongnam-do	Taean	1	2	4	66	-	< 1
Jeollabuk-do	-	-	-	-	-	-	-
Jeollanam-do	Yeosoo	2	7	1	1	159	< 1
Gyeongsangbuk-do	Homigot, Guryongpo, Ullengdo, Dokdo	426	291	190	389	328	318
Gyeongsangnam-do	Tongyeong	778	347	424	173	24	25

Supplementary Table 7. The change of barren status in the eastern coast of South Korea since 2014. The survey data was provided by the Korea Fisheries Resources Agency (FIRA; https://www.fira.or.kr)

Site	Survey area (ha)	Barren area (2014)			Barren area (2017)			Barren area (2019)		
		Area (ratio)			Area (ratio)			Area (ratio)		
		Normal	Mild	Severe	Normal	Mild	Severe	Normal	Mild	Severe
**Gosung**	358.1	147.0ha (41.0%)	167.2ha (46.7%)	43.9ha (12.3%)	160.7ha (44.9%)	156.2ha (43.6%)	41.2ha (11.5%)	190.4ha (53.1%)	112.3ha (31.4%)	55.4ha (15.5%)
Yangyang	468.2	255.3ha (54.6%)	138.7ha (29.6%)	74.2ha (15.8%)	249.1ha (53.2%)	115.2ha (24.6%)	103.9ha (22.2%)	270.1ha (57.7%)	112.0ha (23.9%)	86.1ha (18.4%)
Gangneung	853.0	486.1ha (57.0%)	203.9ha (23.9%)	163.0ha (19.1%)	515.6ha (60.4%)	175.2ha (20.6%)	162.2ha (19.0%)	514.2ha (60.3%)	190.2ha (22.3%)	148.6ha (17.4%)
Donghae	638.4	334.2ha (52.3%)	183.0ha (28.7%)	121.2ha (19.0%)	343.6ha (53.8%)	192.6ha (30.2%)	102.2ha (16.0%)	369.8ha (57.9%)	180.1ha (28.2%)	88.5ha (13.9%)
Samcheok	652.2	345.8ha (53.0%)	159.8ha (24.5%)	146.6ha (22.5%)	367.2ha (56.3%)	121.2ha (18.6%)	163.8ha (25.1%)	404.2ha (62.0%)	153.3ha (23.5%)	94.7ha (14.5%)
**Ulleung do, Dokdo**	794.7	613.1ha (77.1%)	40.3ha (5.1%)	141.3ha (17.8%)	626.1ha (78.8%)	71.2ha (9.0%)	97.4ha (12.2%)	650.6ha (81.9%)	94.6ha (11.9%)	49.5ha (6.2%)
Uljin	1,004.2	548.8ha (54.6%)	236.8ha (23.6%)	218.6ha (21.8%)	503.9ha (50.2%)	299.2ha (29.8%)	201.1ha (20.0%)	494.9ha (49.3%)	248.5ha (24.7%)	260.8ha (26.0%)
Yeongdeok	789.3	356.1ha (45.1%)	147.9ha (18.7%)	285.3ha (36.2%)	371.1ha (47.0%)	183.3ha (23.2%)	234.9ha (29.8%)	378.4ha (47.9%)	182.9ha (23.2%)	228.0ha (28.9%)
**Homigot, Guryongpo**	4,263.1	1,313.7ha (30.8%)	519.9ha (12.2%)	2,429.5ha (57.0%)	1,553.3ha (36.5%)	1,058.9ha (24.8%)	1,650.9ha (38.7%)	1,734.0ha (40.7%)	1,457.0ha (34.2%)	1,072.1ha (25.1%)
Gyeongju	773.2	355.6ha (46.0%)	236.2ha (30.5%)	181.4ha (23.5%)	382.5ha (49.5%)	225.6ha (29.2%)	165.1ha (21.3%)	414.6ha (53.6%)	207.2ha (26.8%)	151.4ha (19.6%)
Ulsan	1,686.7	524.7ha (31.1%)	580.2ha (34.4%)	581.8ha (34.5%)	714.5ha (42.4%)	538.6ha (31.9%)	433.6ha (25.7%)	825.9ha (49.0%)	446.0ha (26.4%)	414.8ha (24.6%)
Busan	1,144.0	612.3ha (53.5%)	372.2ha (32.6%)	159.5ha (13.9%)	664.9ha (58.1%)	310.7ha (27.2%)	168.4ha (14.7%)	695.2ha (60.8%)	279.3ha (24.4%)	169.5ha (14.8%)

Supplementary References

S1. Yoo HI, Park HH, Choi HG. 2009. Marine algal floras and community structures in the vicinity of the Taean power plant in Korea. *Korean J Fish Aqua Sci* 42:387-394. (Korean)

S2. Choi CG. 2008. Algal flora in Hallyeo-haesang National Park, Southern Coast of Korea. *Korean J Fish Aqua Sci* 41:371-380. (Korean)

S3. Park MS, Yoo HI, Heo JS, Kim YD, Choi HG. 2011. Seasonal variation in the marine algal flora and community structure along the Tongyeong Coast, Korea. *Korean J Fish Aqua Sci* 44:732-739. (Korean)

S4. Yoo JS, Park IS, Song YC, Seo Y, Do GY, Lee JW, Ahn JK. 2006. Characteristics and structure of benthic algal community in Pohang new port area. *J Navig Port Res* 30:309-314.

S5. Yoo JS. 2003. Structural characteristics of benthic algal community in the subtidal zone of Yeongil inner and outer bay. *Algae* 18:365-369. (Korean)

S6. Kim YD, Park MS, Yoo HI, Kim SW, Jeong HD, Min BH, Jin HJ. 2012. Characteristics of seasonal variation of subtidal seaweed community structure at three areas in the east coast of Korea. *J Environ Sci Int* 21:1407-1418. (Korean)

S7. Jung SW, Oh YS, Rho HS, Choi CG. 2020. Subtidal marine algal community and endangered species in Dokdo and Ulleungdo, two oceanic islands in the east sea of *Korea. Ocean Sci J* 55:537-547.

S8. Choi CG, Kwon CJ, Kim MK. 2014. Summer marine algal communities at Dokdo, Korea. *J Fish Mar Sci Edu* 26:1037-1043. (Korean)

Codes used in this study

```
#library
library(vegan)
library(ggplot2)
library(dunn.test)
library(gplots)
require(minpack.lm)
require(Hmisc)
require(stats4)
require(dplyr)
library(eulerr)
library(microbiome)
library(microbiomeutilities)
library(RColorBrewer)
library(reshape)
library(Maaslin2)
library(magrittr)
library(scales)
library(grid)
library(reshape2)
library(phyloseq)
library(randomForest)
library(knitr)
library(datasets)
library(caret)
library(pROC)
library(metagenomeSeq)
library(ggsankey)


#Envfit analysis
env_input = read.csv("Genus_colon_0.05_prev_table.csv")

genus = env_input[,17:211]
```

```
meta = env_input[,1:16]

m_genus = as.matrix(genus)

nmds=metaMDS(m_genus, k=2, distance="bray", traymax=50, engine="monoMDS", autotransform = T)

en = envfit(nmds, meta, permutations = 999, strata = NULL, choices=c(1,2), display = "sites", w = weights(nmds), na.rm = TRUE)

en

#NMDS analysis based on Bray-Curtis distance
#Data importing
data <-read.csv("Genus_0.05_envs_prev.table.csv")

nmds=metaMDS(data[,4:374], k=2, distance="bray", traymax=50, engine="monoMDS", autotransform = T)
nmds.ggplot=as.data.frame(scores(nmds, display="sites"))
head(nmds.ggplot)

nmds.ggplot$Group=as.factor(data$Sampling_parts)
head(nmds.ggplot)

p01=ggplot(data=nmds.ggplot, aes(x=NMDS1, y=NMDS2,color=Group)) +
  geom_point(size=2) +
  scale_color_manual(values = c("#1118AC","#F79D00","#9770CC","#005C42"))+
  geom_hline(yintercept = 0, lty="dashed")+
  geom_vline(xintercept = 0, lty="dashed")+
  theme_bw()+
  stat_ellipse(aes(fill=Group), geom = "polygon", level = 0.95, alpha = 0.1, linetype = 2)+
  scale_fill_manual(values = c("#1118AC","#F79D00","#9770CC","#005C42"))+
  theme(panel.grid.major = element_blank(), panel.grid.minor = element_blank())+
  theme(axis.title = element_text(size=15), axis.text = element_text(size=15),
        legend.title = element_text(size=20), legend.text = element_text(size=15))

plot(p01)
```

```
#ANOSIM
otu=data[,4:374]
meta=data[,2:3]
otu.dist = vegdist(otu)
otu.anosim = with(meta, anosim(otu.dist,Sampling_parts))
summary(otu.anosim)

#ADONIS(PERMANOVA)
otu=data[,4:374]
otu.dist=vegdist(otu, distance="bray")
adonis2(otu.dist ~ Sampling_parts*Sampling_parts, data = data, permutations = 999)

#Shannon diversity&Bacterial amounts boxplot

data2 <- read.csv("diversity.csv",header=T,row.names = 1)

p02=ggplot(data2, aes(x=Sampling_parts, y= Shannon_diversity ,fill= Sampling_parts)) +
  geom_boxplot(color = "white")+
  scale_fill_manual(values = c("#1118AC","#F79D00","#9770CC","#005C42"))+
  theme_test()+
  theme(legend.text = element_text(size=15), legend.title = element_text(size=15))+
  theme(axis.title = element_text(size=15), axis.text = element_text(size=15))

p02

p03=ggplot(data2, aes(x=Sampling_parts , y= Bacterial_amounts ,fill= Sampling_parts)) +
  geom_boxplot(color = "white")+
  scale_fill_manual(values = c("#1118AC","#F79D00","#9770CC","#005C42"))+
  theme_test()+
  theme(legend.text = element_text(size=15), legend.title = element_text(size=15))+
  theme(axis.title = element_text(size=15), axis.text = element_text(size=15))

p03
```

```
#Dunn.test
dunn.test(data2$Shannon_diversity, data2$Sampling_parts, method = "bh")
dunn.test(data2$Sampling_parts, data2$Sampling_parts, method = "bh")

#Wilcoxon rank-sum test
wilcox.test(data=data2, Shannon_diversity ~ Sampling_parts)

#Relative Abundance of Dominant species Heatmap

D1 <-read.csv("Genus_0.05_envs_top20_dominant_genus_table.csv", row.names = 1)

D1 <-D1[,02:44]

D1 <- D1+0.001

H=data.matrix(D1)

heatmap.2(log(H),    Rowv = "none", Colv = "none", dendrogram = "none",
          trace="none", key=TRUE,col = colorRampPalette(c("darkblue","darkcyan","green"))(300),
          margins=c(8,8),    cexRow = 0.9, cexCol = 0.9, density.info = "none", key.xlab = "Relative
      Abundance(%)",
          ColSideColors = c(rep("#1118AC",18), rep("#F79D00",18),rep("#9770CC",3),rep("#005C42",4)))


#Correlated with Seawater temperature and microbiota heatmap

D2 <-read.csv("Seawater_microbiota_correlated.csv", row.names = 1)

D2 <- D2+0.001

H=data.matrix(D2)
th=t(H)

#Spearman correlation for dendrograms
```

```
data.dist<-vegdist(H, method = "bray")

data.dist.g <-vegdist(th, method = "bray")

hr <-hclust(as.dist(1-cor(th, method = "spearman")), method = "complete") #Spearman correlation ????��?? clustering(row))

hr2 <-hclust(as.dist(1-cor(H, method = "spearman")), method = "complete") # column


heatmap.2(th,Rowv = as.dendrogram(hr2) , Colv = "none", dendrogram = "row",

          cexRow = 0.7, cexCol = 1, scale = "row", trace = "none", density.info = "none",

          lwid= c(2,8), lhei = c(1,2), margins = c(5,15),

          ColSideColors = c(rep("#440154",6), rep("#433982",6),rep("#30678D",6),rep("#218F8B",6),rep("#36B677",7),rep("#8ED542",6),rep("#FDE725",6),rep("#FC9B25",6)),

          key = TRUE, col = colorRampPalette(c("darkblue","white","red"))(300))


#Neutral model analysis code


spp <- read.csv("p_mild.csv", row.names = 1, header = T)

pool <- read.csv("p_mild.csv", row.names = 1, header = T)

options(warn=-1)


N <- mean(apply(spp, 1, sum))


if(is.null(pool)){

  p.m <- apply(spp, 2, mean)

  p.m <- p.m[p.m != 0]

  p <- p.m/N

} else {

  p.m <- apply(pool, 2, mean)

  p.m <- p.m[p.m != 0]

  p <- p.m/N

}


spp.bi <- 1*(spp>0)

freq <- apply(spp.bi, 2, mean)

freq <- freq[freq != 0]
```

```
C <- merge(p, freq, by=0)
C <- C[order(C[,2]),]
C <- as.data.frame(C)
C.0 <- C[!(apply(C, 1, function(y) any(y == 0))),]
p <- C.0[,2]
freq <- C.0[,3]
names(p) <- C.0[,1]
names(freq) <- C.0[,1]

d = 1/N

##Fit model parameter m (or Nm) using Non-linear least squares (NLS)
m.fit <- nlsLM(freq ~ pbeta(d, N*m*p, N*m*(1-p), lower.tail=FALSE), start=list(m=0.1))
m.ci <- confint(m.fit, 'm', level=0.95)

##Fit neutral model parameter m (or Nm) using Maximum likelihood estimation (MLE)
sncm.LL <- function(m, sigma){
   R = freq - pbeta(d, N*m*p, N*m*(1-p), lower.tail=FALSE)
   R = dnorm(R, 0, sigma)
   -sum(log(R))
}
m.mle <- mle(sncm.LL, start=list(m=0.1, sigma=0.1), nobs=length(p))

##Calculate Akaike's Information Criterion (AIC)
aic.fit <- AIC(m.mle, k=2)
bic.fit <- BIC(m.mle)

##Calculate goodness-of-fit (R-squared and Root Mean Squared Error) @We used@
freq.pred <- pbeta(d, N*coef(m.fit)*p, N*coef(m.fit)*(1-p), lower.tail=FALSE)
Rsqr <- 1 - (sum((freq - freq.pred)^2))/(sum((freq - mean(freq))^2))
RMSE <- sqrt(sum((freq-freq.pred)^2)/(length(freq)-1))

pred.ci <- binconf(freq.pred*nrow(spp), nrow(spp), alpha=0.05, method="wilson", return.df=TRUE)

##Calculate AIC for binomial model
```

```
bino.LL <- function(mu, sigma){
  R = freq - pbinom(d, N, p, lower.tail=FALSE)
  R = dnorm(R, mu, sigma)
  -sum(log(R))
}
bino.mle <- mle(bino.LL, start=list(mu=0, sigma=0.1), nobs=length(p))

aic.bino <- AIC(bino.mle, k=2)
bic.bino <- BIC(bino.mle)

##Goodness of fit for binomial model
bino.pred <- pbinom(d, N, p, lower.tail=FALSE)
Rsqr.bino <- 1 - (sum((freq - bino.pred)^2))/(sum((freq - mean(freq))^2))
RMSE.bino <- sqrt(sum((freq - bino.pred)^2)/(length(freq) - 1))

bino.pred.ci <- binconf(bino.pred*nrow(spp), nrow(spp), alpha=0.05, method="wilson", return.df=TRUE)

##Calculate AIC for Poisson model
pois.LL <- function(mu, sigma){
  R = freq - ppois(d, N*p, lower.tail=FALSE)
  R = dnorm(R, mu, sigma)
  -sum(log(R))
}
pois.mle <- mle(pois.LL, start=list(mu=0, sigma=0.1), nobs=length(p))

aic.pois <- AIC(pois.mle, k=2)
bic.pois <- BIC(pois.mle)

##Goodness of fit for Poisson model
pois.pred <- ppois(d, N*p, lower.tail=FALSE)
Rsqr.pois <- 1 - (sum((freq - pois.pred)^2))/(sum((freq - mean(freq))^2))
RMSE.pois <- sqrt(sum((freq - pois.pred)^2)/(length(freq) - 1))

pois.pred.ci <- binconf(pois.pred*nrow(spp), nrow(spp), alpha=0.05, method="wilson", return.df=TRUE)
```

```
##Results
if(stats==TRUE){
  fitstats <- data.frame(m=numeric(), m.ci=numeric(), m.mle=numeric(), maxLL=numeric(), poisLL=numeric(), Rsqr=numeric(), Rsqr.bino=numeric(), Rsqr.pois=numeric(), RMSE=numeric(), RMSE.bino=numeric(), RMSE.pois=numeric(), AIC=numeric(), BIC=numeric(), AIC.pois=numeric(), BIC.pois=numeric(), N=numeric(), Samples=numeric(), Richness=numeric(), Detect=numeric())
  fitstats[1,] <- c(coef(m.fit), coef(m.fit)-m.ci[1], m.mle@coef['m'], m.mle@details$value, pois.mle@details$value, Rsqr, Rsqr.bino, Rsqr.pois, RMSE, RMSE.bino, RMSE.pois, aic.fit, bic.fit, aic.pois, bic.pois, N, nrow(spp), length(p), d)
  return(fitstats)
} else {
  A <- cbind(p, freq, freq.pred, bino.pred)
  A <- as.data.frame(A)
  colnames(A) <- c('p', 'freq', 'freq.pred', 'bino.pred')
  if(is.null(taxon)){
    B <- A[order(A[,1]),]
  } else {
    B <- merge(A, taxon, by=0, all=TRUE)
    row.names(B) <- B[,1]
    B <- B[,-1]
    B <- B[order(B[,1]),]
  }
  return(B)
}

pre.ci.fit.neutral <- cbind(C,pred.ci[,1:3])

pre.ci.fit.neutral$color <- ifelse(pre.ci.fit.neutral$y < pre.ci.fit.neutral$Lower,'Below prediction',
                                   ifelse(pre.ci.fit.neutral$y > pre.ci.fit.neutral$Upper,'Above prediction','neutral'))

p04<- ggplot(data = pre.ci.fit.neutral, aes(x=log10(x) , y=y, color = color)) +
  geom_line(aes(x=log10(x), y = Lower),size = 1.5, color = "Gray30", linetype = "dashed")+
  geom_line(aes(x=log10(x), y = Upper),size = 1.5, color = "Gray30",linetype = "dashed" )+
  geom_line(aes(x=log10(x), y = PointEst),size = 2,    color = "Gray")+
  scale_color_manual(values = c("Green","DarkBlue","Black"))+
```

```
  geom_point(size = 3)+
  theme_test()+
  theme(panel.grid.minor = element_blank())+
  theme(axis.title = element_text(size=15), axis.text = element_text(size=15))+
  xlab("Log10(Mean Relative Abundance)") + ylab("Occurrence frequency")
p04


#Venn Diagram plot

otu_mat <- read.csv("otu_table.csv", header = T)
tax_mat <-read.csv("tax_table.csv", header = T)
sample_df <-read.csv("sam_table.csv", header = T)
otu_mat <- otu_mat %>% tibble::column_to_rownames("otu")
tax_mat <- tax_mat %>% tibble::column_to_rownames("otu")
sample_df <- sample_df %>% tibble::column_to_rownames("sample")
otu_mat <- as.matrix(otu_mat)
tax_mat <- as.matrix(tax_mat)
OTU = otu_table(otu_mat, taxa_are_rows = TRUE)
TAX = tax_table(tax_mat)
samples = sample_data(sample_df)
myphylo <- phyloseq(OTU, TAX, samples)
myphylo

table(meta(myphylo)$Group, useNA = "always")

pseq.rel <- microbiome::transform(myphylo, "compositional")
meta_states <- unique(as.character(meta(pseq.rel)$Group))
print(meta_states)

for (n in meta_states){
  ps.sub <- subset_samples(pseq.rel, Group == n)
  core_m <- core_members(ps.sub,
                         detection = 0.005,
                         prevalence = 0.00)
```

```
    print(paste0("No. of core taxa in ", n, " : ", length(core_m)))
    list_core[[n]] <- core_m
}

print(list_core)

mycols <- c("#F79D00","#1118AC","#9770CC","#005C42")

myCol <- brewer.pal(4, "Set3")

venn(list_core)
plot(venn(list_core),
        col=c("White"),
        fill = mycols)

#Microbiota dissimilarity analysis (Phyrynx, Gut microbiota and Algal composition)

p_input <-read.csv("pharynx.csv", row.names = 1, check.names = F)
g_input <-read.csv("gut.csv", row.names = 1, check.names = F)
a_input <-read.csv("algal.csv", row.names = 1, check.names = F)

bray_p=vegdist(p_input)
bray_g=vegdist(g_input)
bray_a=vegdist(a_input)

bray_p[upper.tri(bray_p, diag = TRUE)] <- NA
bray_g[upper.tri(bray_g, diag = TRUE)] <- NA
bray_a[upper.tri(bray_a, diag = TRUE)] <- NA

melt.bray.results_p <- melt(as.matrix(bray_p))
melt.bray.results_g <- melt(as.matrix(bray_g))
melt.bray.results_a <- melt(as.matrix(bray_a))

melt.bray.results_p <- na.omit(melt.bray.results_p)
melt.bray.results_g <- na.omit(melt.bray.results_g)
```

```
melt.bray.results_a <- na.omit(melt.bray.results_a)

all_bray_results <- cbind(melt.bray.results_p$value, melt.bray.results_g$value, melt.bray.results_a$value)
names(data.all) <- c("Pharynx_dissimilarity", "Gut_dissimilarity", "Algal_dissimilarity")

p05 = ggplot(data = all_bray_results, aes(x=Algal_dissimilarity, y=Pharynx_dissimilarity)) +
  geom_smooth(linewidth=2, method = lm, se = TRUE, color ="#1118AC")+
  geom_jitter(position = position_jitter(height = 0, width = .1), size = 2, alpha = 0.5) +
  theme_test()+
  theme(panel.grid.minor = element_blank())+
  theme(axis.title = element_text(size=15), axis.text = element_text(size=15))

plot(p05)

#Spearman Correlation

cor.test(all_bray_results$Pharynx_dissimilarity, all_bray_results$Algal_dissimilarity,
         method = "spearman",
         alternative = "greater")

#MaAsLin2 code

input_data <- read.table("sam_table.txt", header = T, sep = "\t", row.names = 1, stringsAsFactors = FALSE)
input_metadata <- read.table("meta_table.txt", header = T, sep = "\t", row.names = 1, stringsAsFactors = FALSE)

fit_data <- Maaslin2(
  input_data, input_metadata, 'masslin_output',
  fixed_effects = c('Seawater_temperature'),
  random_effects = c('Site'),
  min_prevalence = 0,
  min_abundance = 0,
  max_significance = 0.5,
  normalization = 'none',
  plot_scatter = 'FALSE'
)
```

```
# CSS normalization

CSS_input <- read.csv("ko_pathway_table.csv", row.names=1)

metaSeqObject        = newMRexperiment(CSS_input)

metaSeqObject_CSS    = cumNorm( metaSeqObject , p=cumNormStatFast(metaSeqObject) )

read_count_CSS = data.frame(MRcounts(metaSeqObject_CSS, norm=TRUE, log=FALSE))

#RandomForest analysis code

predictors <- t(otu_table(myphylo))
dim(predictors)

response <- as.factor(sample_data(myphylo)$Status)

rf.data <- data.frame(response, predictors)

set.seed(123)

ind <- sample(2, nrow(rf.data), replace = TRUE, prob = c(0.7, 0.3))
train <- rf.data[ind==1,]
test <- rf.data[ind==2,]

# Algorithm Tune (tuneRF)
x <- train[,2:366]
y<- as.factor(train[,1])
seed <- 7
set.seed(seed)
bestmtry <- tuneRF(x, y, stepFactor=1.5, improve=1e-5, ntree=1000)
print(bestmtry)
plot(bestmtry)
```

```
# making model
forest_m <- randomForest(response ~ ., data=train, ntree = 1000, mtry = 13)
forest_m
forest_m$predicted
forest_m$importance

# Prediction & Confusion Matrix - Train data
# focusing at Accuracy, p-value, kappa(normalization of accuracy), 95% CI(confidence interval)
p1 <- predict(forest_m, train)
confusionMatrix(p1, train$response)

# Prediction & Confusion Matrix - test data

p2 <- predict(forest_m, test)
confusionMatrix(p2, test$response)

# Make a data frame with predictor names and their importance
imp <- importance(forest_m)
imp <- data.frame(predictors = rownames(imp), imp)

taxon_table <- tax_table(myphylo)
species <- data.frame(taxon_table[,20])
imp <- cbind(imp, species)

# Order the predictor levels by importance
imp.sort <- arrange(imp, desc(MeanDecreaseGini))
imp.sort$predictors <- factor(imp.sort$predictors, levels = imp.sort$predictors)

imp.sort$Genus <- factor(imp.sort$Genus, levels = imp.sort$Genus)

imp.20 <- imp.sort[1:20, ]
imp.plot <- ggplot(imp.20, aes(x = Genus, y = MeanDecreaseGini)) +
  geom_bar(stat = "identity", fill = "gray30") +
  coord_flip() +
```

```
    theme_test()+
    ggtitle("Most important Top 20 feature")

plot(imp.plot)

#K-fold cross validation of training data

train.x <- train[,2:366]
train.y <- as.numeric(train[,1])

cv <- rfcv(train.x, train.y, cv.fold = 10, ntree = 1000)

#Cross validation value
cv$n.var
cv$error.cv
cv$predicted

cv.error <- data.frame(cv$error.cv)
cv.var <- data.frame(cv$n.var)
cv.data <- cbind(cv.error,cv.var)

cv.plot <- ggplot(cv.data, aes(x= cv.n.var, y= cv.error.cv))+
    geom_line(size=1)+
    geom_point()+
    coord_flip()+
    theme_test()+
    xlab("Number of feature") + ylab("10-fold Cross Validation")

plot(cv.plot)

cv.error

# AUC curves using 10-fold cross validation

set.seed(1)
```

```
flds <- createFolds(rf.data$response, k=10, list=TRUE, returnTrain=FALSE)
str(flds)

experiment <- function(cv_train, cv_test, m) {
  rf <- randomForest(response ~ ., data=cv_train, ntree=100)
  rf_pred <- predict(rf, cv_test, type="response")
  m$acc = c(m$acc, confusionMatrix(rf_pred, cv_test$response)$overall[1])
  rf_pred_prob <- predict(rf, test, type="prob")
  rf_pred <- prediction(rf_pred_prob[,2], test$response)
  m$auc = c(m$auc, performance(rf_pred, "auc")@y.values[[1]])
  return(m)
}

measure = list()
for(i in 1:10){
  inTest <- flds[[i]]
  rf.test <- rf.data[inTest, ]
  rf.train <- rf.data[-inTest, ]
  measure = experiment(rf.train, rf.test, measure)
}

measure

#Accuracy
mean(measure$acc); sd(measure$acc)

#AUC
mean(measure$auc); sd(measure$auc)

#pROC calculate CI with 2000 bootstrapping

rocobj <- plot.roc(rf.train$response, rf.train$rf_pred_prob[,2],
                   percent = TRUE,
                   ci = TRUE,
                   of = "se",
```

```
                              specificities = seq(0, 100, 5),
                              ci.type="shape",
                              ci.col="#1c61b6AA")

rocobj <- plot.roc(rf.train$response, rf.train$rf_pred_prob[,2],
                              ci = TRUE,
                              print.auc = TRUE,
                              specificities = seq(0, 1, 0.05))

lines(smooth(rocobj,
                    method = "fitdistr",
                    density = "lognormal"),
          col = "Black",
          specificities = seq(0, 1, 0.05))

ciobj <- ci.se(rocobj, boot.n=5000, cof.level=0.95)
plot(ciobj, type = "shape", col = "Gray30",
        specificities = seq(0, 1, 0.05))

plot(ciobj, type = "bar",
        specificities = seq(0, 1, 0.01))


#Volcano plot analysis

rd = read.csv("metabolite_table.csv")
names(rd)[2]
in.data <- rd[,16:3951]
in.data <- in.data+1

Mild <- log2(apply(in.data[1:30,],2,mean))

Severe <- log2(apply(in.data[31:49,],2,mean))

Fold.change.value <- Mild - Severe
```

```
names(rd)[2]
pheno<-rd[,"Barren"]
analysis_func<-function(t_data,t_pheno){
  t_data<-apply(t_data,2,as.numeric)
  result<-apply(t_data,2,function(data,pheno){
    wilcox.test(data~pheno)
  },t_pheno)
  result2<-data.frame(t(sapply(result,function(data){
    c(data$statistic,data$p.value)
  })),stringsAsFactors = F)
  colnames(result2)<-c("Statistic","p-value")
  return(result2)
}
result_out<-analysis_func(rd[,-1:-2],pheno)

q_value<-p.adjust(result_out$`p-value`,method="BH")

result_out2<-cbind(result_out,q_value)

Fold.change.data <- cbind(Fold.change.value, result_out2[,2:3])

p07 <- ggplot(data=Fold.change.data, aes(x=Fold.change.value, y=-log10(pval), color=group))+
  geom_point(size=3) +
  scale_color_manual(values=c("#6F8200","#861700","gray"))+
  theme_test() +
  geom_vline(xintercept=c(-0.5,0,0.5), col="darkgray", linetype = "dashed", size=1) +
  geom_hline(yintercept=c(-log10(0.5)), col="darkgray", linetype = "dashed",size=1) +
  coord_cartesian(xlim = c(-6,6)) # ?? ��??

plot(p07)

#Sankey diagram plotting
d <- read.csv("k_gene_input.csv")
```

```
df <- d %>%
  make_long(Dominant, gene, bacteria)
df

pl <- ggplot(df, aes(x = x
                     , next_x = next_x
                     , node = node
                     , next_node = next_node
                     , fill = factor(node)
                     , label = node))
pl <- pl +geom_sankey(flow.alpha = 1
                      , node.color = "black"
                      ,show.legend = FALSE)
pl <- pl +geom_sankey_label(size = 3, color = "black", fill= "white", hjust = -0.5)
pl <- pl +    theme_bw()
pl <- pl + theme(legend.position = "none")
pl <- pl +    theme(axis.title = element_blank()
                  , axis.text.y = element_blank()
                  , axis.ticks = element_blank()
                  , panel.grid = element_blank())
pl <- pl + scale_fill_viridis_d(option = "inferno")
pl
```
