# Supplementary material for: Alterations in sea urchin (Mesocentrotus nudus) microbiota and their potential contributions to host according to barren severity
Source: NPJ Biofilms Microbiomes. 2023 Oct 31;9:83. doi: 10.1038/s41522-023-00450-z (PMC10618176; doi:10.1038/s41522-023-00450-z)
Supplement: Supplementary file 2 — Reporting summary [file 41522_2023_450_MOESM2_ESM.pdf]

nature portfolio

Corresponding author(s): Bong-Soo Kim

Last updated by author(s): Aug 16, 2023

# Reporting Summary

Nature Portfolio wishes to improve the reproducibility of the work that we publish. This form provides structure for consistency and transparency in reporting. For further information on Nature Portfolio policies, see our Editorial Policies and the Editorial Policy Checklist.

## Statistics

For all statistical analyses, confirm that the following items are present in the figure legend, table legend, main text, or Methods section.

| n/a | Confirmed | |
|---|---|---|
| ☐ | ☒ | The exact sample size (*n*) for each experimental group/condition, given as a discrete number and unit of measurement |
| ☐ | ☒ | A statement on whether measurements were taken from distinct samples or whether the same sample was measured repeatedly |
| ☐ | ☒ | The statistical test(s) used AND whether they are one- or two-sided<br>*Only common tests should be described solely by name; describe more complex techniques in the Methods section.* |
| ☐ | ☒ | A description of all covariates tested |
| ☐ | ☒ | A description of any assumptions or corrections, such as tests of normality and adjustment for multiple comparisons |
| ☐ | ☒ | A full description of the statistical parameters including central tendency (e.g. means) or other basic estimates (e.g. regression coefficient) AND variation (e.g. standard deviation) or associated estimates of uncertainty (e.g. confidence intervals) |
| ☐ | ☒ | For null hypothesis testing, the test statistic (e.g. *F*, *t*, *r*) with confidence intervals, effect sizes, degrees of freedom and *P* value noted<br>*Give P values as exact values whenever suitable.* |
| ☒ | ☐ | For Bayesian analysis, information on the choice of priors and Markov chain Monte Carlo settings |
| ☐ | ☒ | For hierarchical and complex designs, identification of the appropriate level for tests and full reporting of outcomes |
| ☐ | ☒ | Estimates of effect sizes (e.g. Cohen's *d*, Pearson's *r*), indicating how they were calculated |

*Our web collection on statistics for biologists contains articles on many of the points above.*

## Software and code

Policy information about availability of computer code

| | |
|---|---|
| Data collection | The annual report on population dynamics data of sea urchins and the survey report of urchin barren located in the South Korean coast were provided by the Korea Fisheries Resources Agency (FIRA) and Korean Statistical Information Service (KOSIS). The collected data was summarized in Supplementary Tables S5, S6, and S7. 16S rRNA amplicon sequencing data was obtained using the MiSeq system. The relative bacterial amounts was estimated by quantitative real-time PCR based on the 16S rRNA gene. |
| Data analysis | Codes used in this study are provided in the supplemental information associated with this manuscript. |

For manuscripts utilizing custom algorithms or software that are central to the research but not yet described in published literature, software must be made available to editors and reviewers. We strongly encourage code deposition in a community repository (e.g. GitHub). See the Nature Portfolio guidelines for submitting code & software for further information.

## Data

Policy information about availability of data

All manuscripts must include a data availability statement. This statement should provide the following information, where applicable:

- Accession codes, unique identifiers, or web links for publicly available datasets
- A description of any restrictions on data availability
- For clinical datasets or third party data, please ensure that the statement adheres to our policy

The sequence reads obtained from this study are available in the EMBL SRA database under the study number PRJEB57350 (http://ebi.ac.uk/ena/browser/view/PRJEB57350).

## Research involving human participants, their data, or biological material

Policy information about studies with human participants or human data. See also policy information about sex, gender (identity/presentation), and sexual orientation and race, ethnicity and racism.

| | |
|---|---|
| Reporting on sex and gender | N/A |
| Reporting on race, ethnicity, or other socially relevant groupings | N/A |
| Population characteristics | N/A |
| Recruitment | N/A |
| Ethics oversight | N/A |

Note that full information on the approval of the study protocol must also be provided in the manuscript.

# Field-specific reporting

Please select the one below that is the best fit for your research. If you are not sure, read the appropriate sections before making your selection.

☐ Life sciences ☐ Behavioural & social sciences ☒ Ecological, evolutionary & environmental sciences

For a reference copy of the document with all sections, see nature.com/documents/nr-reporting-summary-flat.pdf

# Ecological, evolutionary & environmental sciences study design

All studies must disclose on these points even when the disclosure is negative.

| | |
|---|---|
| Study description | To understand microbiota in sea urchin according to diet availability by kelp deforestation, we analyzed the microbiota in the pharynx and gut of Mesocentrotus nudus, a common sea urchin along the coast of South Korea, and the microbiota of their habitats (sand and seawater) collected from eight barren regions (five mild and three severe barren regions) in South Korea. |
| Research sample | Seven sea urchins were randomly collected from eight sites because the minimum collected number of sea urchins among the sampling sites was seven. We separated pharynx and gut from the collected samples. One sea urchin sample in each site (except site G) was excluded owing to failed sequencing library preparation. Three seawater and four sand samples were collected from one severe barren region and two mild barren regions. Thus, a total of 105 sequence data obtained from samples of sea urchins (49 pharynx and 49 gut) and habitats (3 seawater and 4 sand) were analyzed after removing potential contaminants based on sequences detected in 27 negative controls (from sampling to library preparation processes). |
| Sampling strategy | Sampling sites were selected based on the annual report on population dynamics data of sea urchins and the survey report of urchin barren located in the South Korean coast were provided by the Korea Fisheries Resources Agency (FIRA) and Korean Statistical Information Service (KOSIS). The collected data was summarized in Supplementary Tables S5, S6, and S7. Sea urchins (n=7) were randomly collected from each site because the minimum collected number of sea urchins among the sampling sites was seven. Sea urchin habitat samples (sand and seawater) were collected from three sites (Ulleung do, Dokdo, and Guryongpo) that were selected according to barren severity. As sea urchins were collected from two different sand regions in Guryongpo, two sand samples (surrounding habitat for sea urchins) were collected from this site. |
| Data collection | Sampling sites were selected based on the annual report on population dynamics data of sea urchins and the survey report of urchin barren located in the South Korean coast were provided by the Korea Fisheries Resources Agency (FIRA) and Korean Statistical Information Service (KOSIS). The barren status was determined based on the cover degree of crustose corallines in the survey region using hyperspectral aerial imaging by the FIRA. 16S rRNA amplicon sequencing data was obtained using the MiSeq system. The relative bacterial amounts was estimated by quantitative real-time PCR based on the 16S rRNA gene. |

| | |
|---|---|
| Timing and spatial scale | Sea urchin and habitat samples were collected between June and July 2021. |
| Data exclusions | One sample in each site (except site G) was excluded owing to failed sequencing library preparation (from excise pharynx and gut tissue to sequencing library preparation). For comparison between gut and pharynx in each sea urchin, we excluded partially obtained sample data (only obtained gut or pharynx in each sample). |
| Reproducibility | The sequence reads obtained from this study are available in the EMBL SRA database. Information about the barren status and biomass of sea urchins are included in our supplemental information. |
| Randomization | Sea urchins (n=7) were randomly collected from each site because the minimum collected number of sea urchins among the sampling sites was seven. The influence of covariates on microbiota was analyzed using the EnvFit model. We used the MaAsLin2 to adjust for confounding factors when performing the correlation analysis. |
| Blinding | N/A |

Did the study involve field work? ☐ Yes ☒ No

# Reporting for specific materials, systems and methods

We require information from authors about some types of materials, experimental systems and methods used in many studies. Here, indicate whether each material, system or method listed is relevant to your study. If you are not sure if a list item applies to your research, read the appropriate section before selecting a response.

## Materials & experimental systems

| n/a | Involved in the study |
|---|---|
| ☒ | ☐ Antibodies |
| ☒ | ☐ Eukaryotic cell lines |
| ☒ | ☐ Palaeontology and archaeology |
| ☐ | ☒ Animals and other organisms |
| ☒ | ☐ Clinical data |
| ☒ | ☐ Dual use research of concern |
| ☒ | ☐ Plants |

## Methods

| n/a | Involved in the study |
|---|---|
| ☒ | ☐ ChIP-seq |
| ☒ | ☐ Flow cytometry |
| ☒ | ☐ MRI-based neuroimaging |

# Animals and other research organisms

Policy information about studies involving animals; ARRIVE guidelines recommended for reporting animal research, and Sex and Gender in Research

| | |
|---|---|
| Laboratory animals | N/A |
| Wild animals | Mesocentrotus nudus, a common sea urchin along the coast of South Korea was collected from barren regions. |
| Reporting on sex | N/A |
| Field-collected samples | The annual report on population dynamics data of sea urchins and the survey report of urchin barren located in the South Korean coast were provided by the Korea Fisheries Resources Agency (FIRA) and Korean Statistical Information Service (KOSIS). The collected data was summarized in Supplementary Tables S1, S5, S6, and S7. |
| Ethics oversight | The Korean Ministry of Ocean and Fisheries |

Note that full information on the approval of the study protocol must also be provided in the manuscript.
